# New frontier of hybrid energy storage devices: evolution of capacitive mechanisms and escalation of nanocomposite materials

**DOI:** 10.1039/d5ra05583k

**Published:** 2025-10-14

**Authors:** Salman Farsi, Mushfiqur Rahman, Thuhin K. Dey, A. J. Saleh Ahammad, Mamun Jamal

**Affiliations:** a Department of Materials Science & Engineering, Faculty of Electrical Engineering, Khulna University of Engineering & Technology Khulna Bangladesh; b Department of Leather Engineering, Faculty of Mechanical Engineering, Khulna University of Engineering & Technology Khulna Bangladesh; c Department of Chemistry, Jagannath University Dhaka 1100 Bangladesh; d Department of Chemistry, Faculty of Science & Humanities, Khulna University of Engineering & Technology Khulna Bangladesh mamun.jamal@chem.kuet.ac.bd; e School of Engineering, RMIT University VIC 3001 Australia

## Abstract

The growing popularity of portable electronic devices has led to a high demand for advanced energy storage technology, driven by advancements in power generation, electrification, and transportation. Hybrid energy storage devices offer high energy density, wide potential windows, rapid charging, long cycle life, and flexibility. Nanostructured materials further enhance device performance through synergistic effects. This review compiles a comprehensive range of materials, showcasing their evolution from traditional to advanced forms based on enhanced properties for hybrid energy storage devices. It also critically examines the mechanisms specific to material groups, informed by research advancements. Along with this, we critically describe the adjacent mechanisms that are possessed by individual groups of materials from the evolution phenomena of research progress. Future research scope has also been discussed with focus on the potential of new nanocomposite materials in enhancing capacitive mechanisms.

## Abstract 2

The growing popularity of portable electronic devices has led to a high demand for advanced energy storage technology, driven by advancements in power generation, electrification, and transportation.

## Graphical abstract

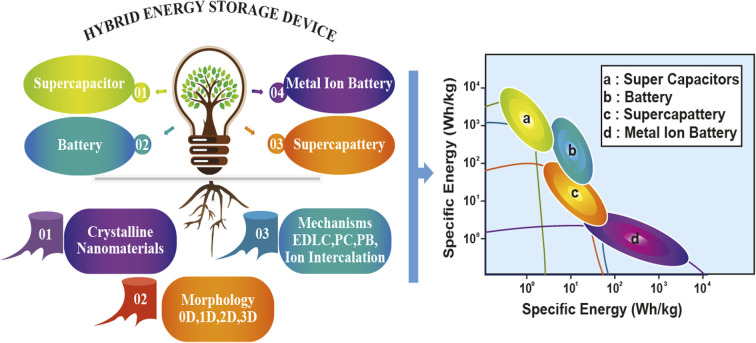

## 1. Introduction

The global economy is rapidly expanding, fossil fuels are depleting, and accelerating environmental deterioration is putting enormous burden on the planet. In order to effectively regulate this, new energy conversion and storage technologies, as well as sustainable, clean, and efficient energy sources, are required. As a result, electrochemical supercapacitors, batteries and super-capatteries are the most often utilized energy storage technologies.^1^ However, batteries have a slow power delivery or uptake rate, making them unable to meet faster and higher-power energy demands. In this scenario, a supercapacitor is created to store and release energy for electrical applications. Supercapacitors are also projected to have a power density comparable to ordinary capacitors, bridging the gap in terms of specific energy and power density between batteries and traditional capacitors.^2^ Rechargeable batteries have greater storage capacity than supercapacitors, which are superior in terms of power, energy efficiency, and cycle life. Several hybrid devices, such as lithium-ion capacitors, redox capacitors and pseudocapacitors, have been developed as a result of their complementary advantages.^3^ Although these hybrids do not retain charge in the same way that a capacitor does, the word capacitor in their titles has led to the misapplication of capacitance as a performance metric.^4^ Although the phrase ‘lithium-ion capacitor’ first arose in 2007; a combination of capacitive and lithium storage electrodes was mentioned recently.^5–7^ Because of its strong ties to lithium-ion batteries, lithium-ion capacitor research and development have kept pace with those of other ion capacitors.^8^ Supercapattery, however, was rarely mentioned in the literature prior to 2015. Curiosity and study of new and superior electrochemical energy storage methods, materials and technologies beyond supercapacitors and rechargeable batteries have fueled recent growth in interest.^9^

The other, more basic cause has to do with pseudocapacitance, which has been incorrectly utilized to explain the behavior of several novel transition metal compounds capable of Nernstian storage.^10^ There is growing concern regarding the above-mentioned mentioned hybrid devices, which are no longer categorized strictly as capacitors or supercapacitors, due to their charge storage mechanisms resembling those of rechargeable batteries. This similarity can lead to confusion with true supercapacitors, particularly in terms of their fundamental principles and commercial development. The term supercapattery offers a unified conceptual framework for studying, comparing, and communicating about these hybrid electrochemical energy storage systems. It is a relatively new term that is gradually gaining recognition within the electrochemical energy storage community.^11,12^ Metal-ion capacitors such as Li-ion and Na-ion capacitors behaves like supercapattery, where it integrate a battery-type electrode that stores charge through faradaic redox reactions with a capacitive electrode that relies on electrostatic charge accumulation, ultimately combining the benefits of both batteries and supercapacitors.^13^

Thereby, in this study, a comprehensive review of the literature on the evolution of processes and material escalation in energy storage devices is presented. Thus, a new frontier opens up to the researchers to develop mechanisms based on specific materials performances and nanocomposite materials, bringing about new blessings on energy storage devices that are also ranked based on the performance study. Finally, we incorporate the focus of future research prospects *via* new nanocomposite materials contributions and evolution on capacitive mechanisms. This analysis highlights current issues in energy storage device design, development, and future research prospects.

## 2. Evolution of capacitive mechanisms

**Fig. 1:**
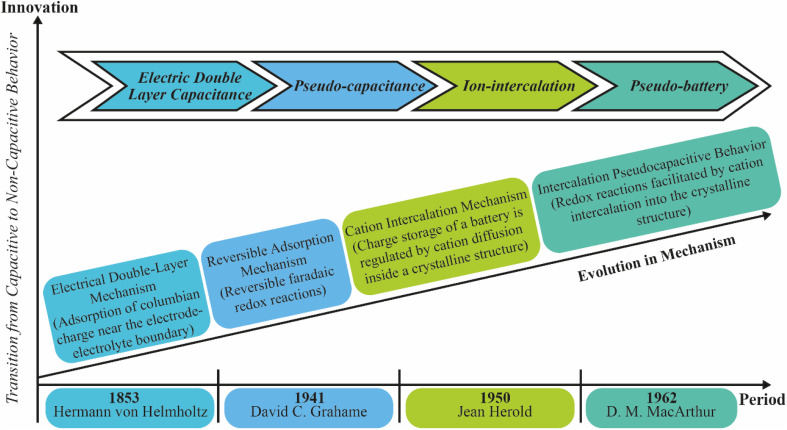
Evolution scenario of energy reservoir mechanisms including innovative capacitive revolution according to the period.

**Fig. 2:**
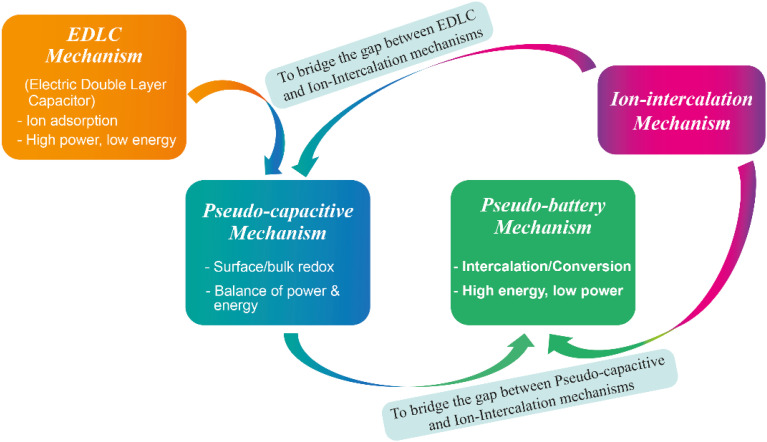
Schematic illustration of the progress in capacitive charge-storage mechanisms, showing the underlying reaction processes and associated performance characteristics.

Researchers have identified electrochemical energy storage mechanisms as complementary options for energy harvesting across various scales, often proving more effective than widely adopted methods like pumped hydroelectric storage. These mechanisms offer high energy and power densities, long cycle life, and the potential for commercial cost-effectiveness.^10^ While metal-ion intercalation systems provide high energy capacities and electric double-layer capacitors (EDLCs) deliver excellent power capabilities and long cycle life, each mechanism alone falls short of meeting all the criteria required for successful commercialization, despite notable advancements in both areas over the past two decades.^14^Fig. 1 highlights a chronological development of different electrochemical storage mechanisms. To address this, the evolution of energy storage mechanisms has been necessarily and preliminarily demonstrated, in which such mechanisms are categorized into four different segments: (i) EDLC, (ii) pseudo-capacitance, (iii) pseudo-battery, and (iv) metal-ion-intercalation, which can combine the merits of one mechanism with those of others into one device. Fig. 2 describes how pseudocapacitive processes bridge the gap between conventional capacitors and battery-type systems.

### 2.1 Electric double layer capacitance

The first revolutionary energy storage mechanism is electric double layer capacitance (EDLC) due to adsorption of columbian charge near the electrode–electrolyte boundary.^14,15^ The electrical double layer mechanism is mainly an electrostatic and electrochemical process in which different models have been proposed for the electrical double-layer mechanism and its structural phenomenon. The three most prominent models are: (i) the Helmholtz double-layer model, (ii) the Gouy–Chapman model, and (iii) the Stern model. Helmholtz hypothesized that the double layer was a quasi-two-dimensional model that consisted of two arrays of opposite charges in which distance separation was about the atomic size. The Helmholtz model is not appropriate for complex systems because ions on the electrolyte solution side are dynamic, but due to thermal changes that rely on electrostatic forces between the ions and the charged metal. Subsequently, an evolution of this theory was carried out by Gouy and Chapman, with some more advances on counter ions. It was pointed out that the counter ions become conjugated within a three-dimensional structural phenomenon in which six electron charges become conjugated with the metal surfaces, appearing as a densely distributed cation and anion stream. An incorrect fact with this model is the local field surrounding the electrode surface, and further prediction is of higher capacitance. The rate of change of the net ionic charge on the electrolyte side of the metal – electrolyte interface is defined later, in which charges support the potential difference across the interface.

**Fig. 3:**
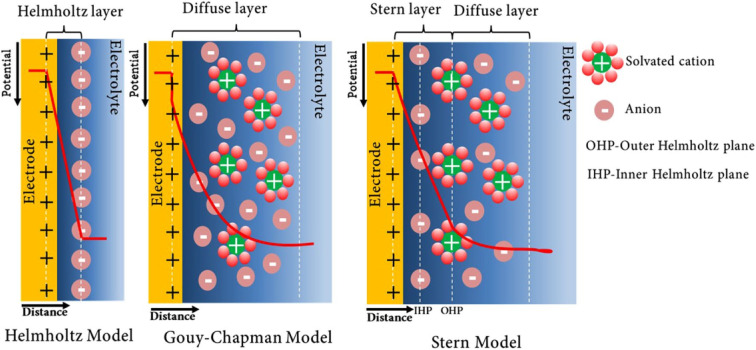
Schematic representation of a double-layer of positive ions in the electrode and solvated cation ions in the liquid medium, detached by a layer of polarized solvent molecules and representation of EDLC configurations: Helmholtz model, Gouy–Chapman model and Gouy–Chapman–Stern model.^14^ [Reproduced from ref. 14 with permission from Springer, copyright 2025].

The overestimation of the higher double-layer capacitance in the Gouy–Chapman hypothesis was improved by Stern. The ion distribution on the inner layer was determined using Stern's model, owing to the adsorption of ions using Langmuir's adsorption isotherm. The diffuse layer containing the scattered ionic charges was regarded as the region between the inner layer and the electrolyte solution (Fig. 3).^14^ Bockris, Devanathan, and Muller developed a model in the 19th century that included the solvent action. It was proposed that due to the electrode's charge, dipoles of the water molecules become aligned, results a layer of water marks at the electrode surface within the inner Helmholtz plane.^16^ Consequently, the electric double layer consists of two opposing charge layers: one embedded within the lattice structure of the electrode surface, and the other formed by oppositely charged, dissolved, and solvated ions from the electrolyte. The two levels are divided by a monolayer of solvent molecules, such as water molecules in the case of water as a solvent, known as the inner Helmholtz plane (IHP). Solvent molecules attach to the surface of the electrode *via* physical adsorption, separating oppositely polarized ions, and can be idealized as a molecular dielectric. Because there is no charge transfer between the electrode and electrolyte during the process, the forces that produce adhesion are physical forces, such as electrostatic forces. In the outer Helmholtz plane, the intensity of counter-charges corresponds to the amount of charge in the electrode (OHP). As a fundamental capacitive mechanism, it represents the initial advancement in hybrid energy storage systems, wherein electrostatic charge separation at the electrode–electrolyte interface facilitates rapid and reversible energy storage, establishing the groundwork for advanced nanostructured materials to improve device performance.

### 2.2 Pseudo-capacitance

Secondly, pseudocapacitance arises when the electric double layer is penetrated by ions adsorbed from the electrolyte. This type of capacitance stores electrical energy through reversible faradaic redox reactions occurring at the surface of suitable electrode materials. In this process, only one electron per charge unit contributes to the pseudocapacitance, involving charge transfer between the electrode and the electrolyte, initiated by an adsorbed and de-solvated ion-illustrated on the left side of the figure below. These fast and reversible processes, such as redox reactions, ion intercalation, or electrosorption, enable faradaic charge transfer without forming chemical bonds. That is, no chemical reaction occurs between the adsorbed ion and the electrode atoms; only electron transfer takes place.^17^

Several types of faradaic reactions may occur at the electrode surface, generally classified into three main types: (i) reversible adsorption (*e.g.*, hydrogen adsorption on gold or platinum); (ii) redox reactions involving transition metal oxides (*e.g.*, RuO_2_), and (iii) reversible electrochemical doping and de-doping in conductive polymer-based electrodes.^1^ Although it is known since the 19th century that the conducting polymers showed pseudo-capacitance for super-capacitor applications through doping and de-doping of the polymer backbone and for sustaining charge neutrality that may result from intercalation and de-intercalation of electrolyte ions through the polymer electrodes, but the concept were harnessed during the 20th century.^18^ Pseudo-capacitors containing conducting polymers and doping/de-doping processes are associated with charge/discharge processes. Organic polymers can be doped in two ways—p-doping (oxidation) and n-doping (reduction)—both of which can significantly enhance the polymer's conductivity, even up to metallic levels. In p-doping, the polymer undergoes partial oxidation, with counter anions (A^−^) inserted to maintain electrical neutrality (as shown in eqn (1)). Conversely, n-doping involves partial reduction of the polymer, accompanied by the insertion of counter cations (M^+^) to preserve charge balance (as illustrated in eqn (2)).

1 *P*_m_ − *x*e^−^ + *x*A^−^ < charge/discharge > *P*_m_^*x*+^ A_*x*_^−^

**Fig. 4:**
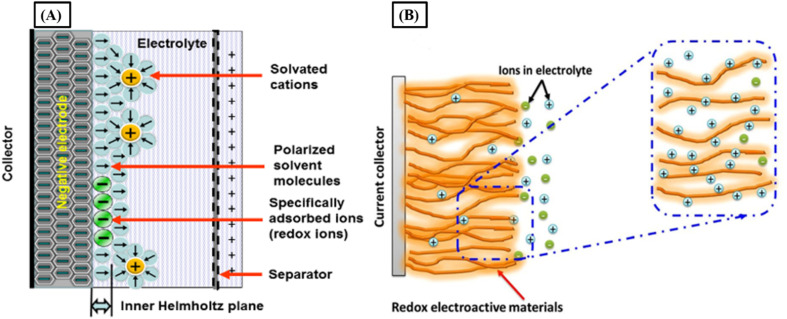
Schematic illustration of a double-layer with specifically adsorbed ions which have gave up their charge to the electrode to occur the faradaic charge transfer of the pseudo-capacitance (left side) and simplified view of the charge storage mechanisms in surface redox (right side).^14,20^ [Reproduced from ref. 14 and 20 with permission from Springer, copyright 2025].

2 *P*_m_ + *x*e^−^ + *x*M^+^ < charge/discharge > *P*_m_^*x*−^ M_*x*_^+^

where *x* is the number of charges transferred and *m* is the polymerization degree.^19^ As part of the continuous evolution in hybrid energy storage systems, it incorporates surface-controlled faradaic processes, where rapid and reversible redox reactions at the electrode–electrolyte interface improve the capacitance significantly through various mechanisms such as ion adsorption, intercalation, and electrochemical doping. It made the groundwork for the future use of high-performance pseudocapacitive materials such as conducting polymers and metal oxides (Fig. 4).^14,20^

### 2.3 Pseudo-battery

The pseudo-battery represents a novel class of charge storage mechanisms that utilizes the intercalation and de-intercalation of cations (*e.g.*, Li^+^, Na^+^, *etc.*) while not being limited by the diffusion of cations within the crystal structure of charge transfer materials (Fig. 4). This mechanism merges the benefits of both batteries and supercapacitors by enabling charge storage without the typical diffusion constraints.

According to Yonggang Wang *et al.*, the kinetics of pseudo-battery behavior closely resemble those of conventional pseudocapacitive systems, yet the electrode characteristics mirror those of battery-type electrodes, where charge storage occurs within a narrow potential window.^21^ In the literature, this mechanism is often referred to as “intercalation pseudocapacitive behavior”, indicating its kinetics are akin to linear, diffusion-independent pseudocapacitive systems. The underlying electrochemical process remains characteristic of battery-type electrodes-specifically, redox reactions facilitated by cation intercalation into the crystalline structure of active materials.

The terminology “pseudo-capacitance” is used to describe some oxide materials (RuO_2_, MnO_2_) or conducting polymer materials (PANI, PPy *etc.*) that have the electrochemical sign of a capacitive electrode (such as carbon based materials) it means a linear relationship between the charge stored and the width of the potential window and even though charge storage comes from different reaction mechanisms. Misunderstanding for readers because the concept of “capacitance” cannot relate to faradaic behavior, whereas “capacity” is the most suitable and significant.^22^

Although scientists have clearly described how different energy storage materials work, some confusion still exists. Many materials that behave like batteries—such as Ni(OH)_2_;^23^ have been incorrectly described as pseudocapacitive in research papers. This leads to misunderstandings about how they actually store energy. For instance, MnO_2_ often shows a rectangular-shaped curve in tests (called cyclic voltammetry), which looks like a pseudocapacitor, but it doesn't truly work the same way. Battery-type materials like Ni(OH)_2_ behave very differently. This confusion also applies to materials like cobalt oxides or hydroxides,^24^ and even mixtures like nickel–cobalt oxides. According to Han Shao, several other materials—such as NiO, Co_3_O_4_, Ni(OH)_2_, and CoHPO_4_—have also been wrongly labeled as pseudocapacitive, which is technically incorrect.^25^

**Fig. 5:**
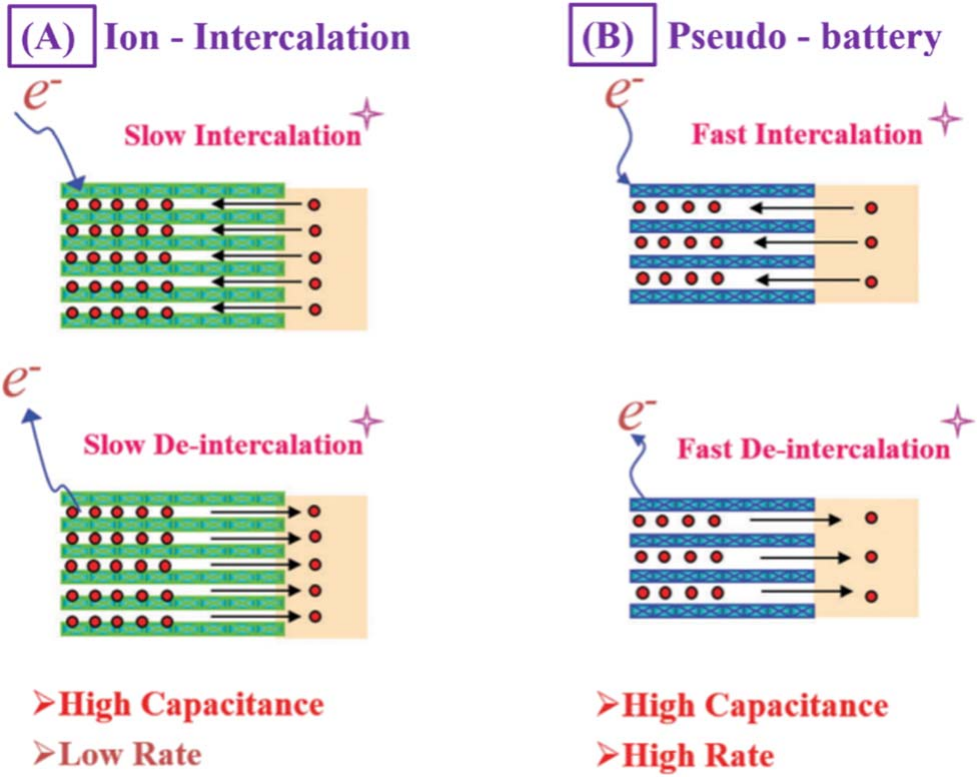
Schematic view of different charge storage mechanisms (A) charge storage mechanism of rechargeable battery that is ion-intercalation; (B) charge storage mechanism of fast intercalation pseudo-battery.^21^ [Reproduced from ref. 21 with permission from The Royal Society of Chemistry, copyright 2025].

Such materials do not have the capacitive performance of carbon-based materials, such as rectangular CV and linear charge–discharge graphs. These faradaic reactions, on the other hand, are driven by diffusion and absorption on the surface of the electrode, rather than the intercalation/deintercalation mechanism found in metal ion batteries. ‘Pseudo’ signifies almost or approaching, therefore, these materials should be named pseudo-battery-type materials since they have battery-like activity and yet no intercalation or massive structure changes created by alloying and conversion that as shown in Fig. 5. One of the most common pseudo-battery-type materials are metal oxides and phosphates, and their storing mechanisms are described in more detail.^26^

The distinction between pseudo-capacitive and battery behavior is more evident and direct in the CV characteristics. We think that our analysis may give a new frontier on this issue and enable readers to properly present their electrodes. As a part of continuous development in hybrid energy storage systems, the pseudo-battery concept fills the gap between batteries and supercapacitors by joining surface and diffusion-controlled redox reactions, resulting in elevated energy and power density.

### 2.4 Ion-intercalation

In general, materials that store energy through metal-ion intercalation (inserting metal ions like Li^+^ into layered structures) are known for their good cycle stability-meaning they can be charged and discharged many times without degrading. However, their theoretical energy capacity is relatively low. In contrast, two other types of charge storage mechanisms can offer much higher capacity, but they suffer from large volume changes during charging and discharging, which reduces their long-term stability and energy efficiency.^27^

In traditional metal-ion batteries, charging and discharging happen through the intercalation and de-intercalation of metal ions.^28^ For example, in a LiCoO_2_–graphite battery, when charging, lithium ions (Li^+^) move out of the layered structure of the LiCoO_2_ cathode, forming CoO_2_. During discharge, the lithium ions return, reversing the process. The charge storage of a battery is regulated by cation diffusion inside a crystalline structure, which is shown in Fig. 4. In addition to the intercalation process outlined above, the mechanisms involved in all rechargeable batteries include “phase-transformation” or alloying reactions. We simply use the intercalation process in the metal-ion battery as an example here to show how a super-capacitor's charge storage mechanism differs from that of a rechargeable battery. Intercalation with minimal crystallographic phase changes: charge storage is usually accompanied by crystallographic phase changes in many metal ion intercalation materials.^21^

The ability of certain materials to easily take in and release metal ions depends heavily on their open crystal structures, chemical makeup, ion concentration, and particle shape. These factors all influence how well a material performs in a battery. During each charge and discharge cycle in a metal-ion battery, the number of metal ions inside the electrodes changes a lot, often leading to structural transformations in the material. These changes can include:

Order-disorder transitions (where atoms or ions become more randomly arranged), two-phase reactions (where different crystal structures exist at once and an interface moves through the material), and phase shifts in the crystal structure itself. While the chemical composition of an electrode determines the voltage range it operates in, the crystal structure affects the shape of the voltage curve as metal ions move in and out. In many key intercalation materials, vacancy clusters-groups of missing atoms or ions in the crystal-help metal ions move through the structure more easily. As a metal ion is given to an already metal ion-rich host, this process derives from the unique crystallographic characteristics of the host, resulting in a significant drop in the metal ion diffusion coefficient.^29^ This ultimate evolutionary phase encompasses ion intercalation in layered or tunnel-structured electrode materials, wherein reversible insertion without structural degradation provides improved cycle stability, capacity retention, and hybridized charge storage performance for next-generation energy devices.

### 2.5 Real-time and practical applications of hybrid capacitive mechanisms spectroscopy

Electrochemical impedance spectroscopy (EIS) delivers information that changes with frequency, shedding light on charge movement, ion diffusion, and events at interfaces. This information is crucial for identifying the fundamental energy storage process. For electric double-layer capacitors (EDLCs), the EIS Nyquist plot usually features a line that is almost vertical in the low-frequency part. This shape points to purely capacitive behavior driven by electrostatic charge separation, with no chemical reactions involved.^30^ The plot also reveals a low equivalent series resistance and a very small semicircle, confirming rapid ion adsorption and desorption. In contrast, pseudocapacitive materials show a flattened semicircle in the high to middle frequency range, caused by resistance from charge transfer in chemical reactions. At low frequencies, this is followed by a slanted line. This combined pattern suggests energy storage comes from both surface-based redox reactions and capacitive ion adsorption. Meanwhile, EIS data for battery-like systems often display a larger semicircle, indicating slower reaction kinetics, and a Warburg diffusion tail at low frequencies, a sign of ions moving into the bulk material. This helps tell them apart from pseudocapacitors, where charge transfer is quicker and confined to the surface. For electrodes that store charge through ion insertion into the crystal structure, EIS highlights strong Warburg impedance and extended diffusion traits, as the movement of ions in and out of the lattice controls the speed. The semicircle related to charge transfer resistance is typically larger, matching the nature of reactions that involve the bulk material.

Therefore, by examining Nyquist plots and frequency responses, EIS can effectively separate capacitive (EDLC), surface-reaction-based (pseudocapacitive), and diffusion-controlled (battery-like or ion-insertion) charge storage mechanisms.^31^ When EIS is used during device operation (*operando*), it becomes possible to link changes in resistance, diffusion, and charge-transfer dynamics to the applied voltage.^32^ Together, these advanced analysis techniques offer vital insights into the operating mechanisms—insights that studies conducted on inactive materials cannot provide. They are essential for connecting the discovery of new materials to the practical improvement of device performance.

## 3. Hybrid capacitive mechanism

**Fig. 6:**
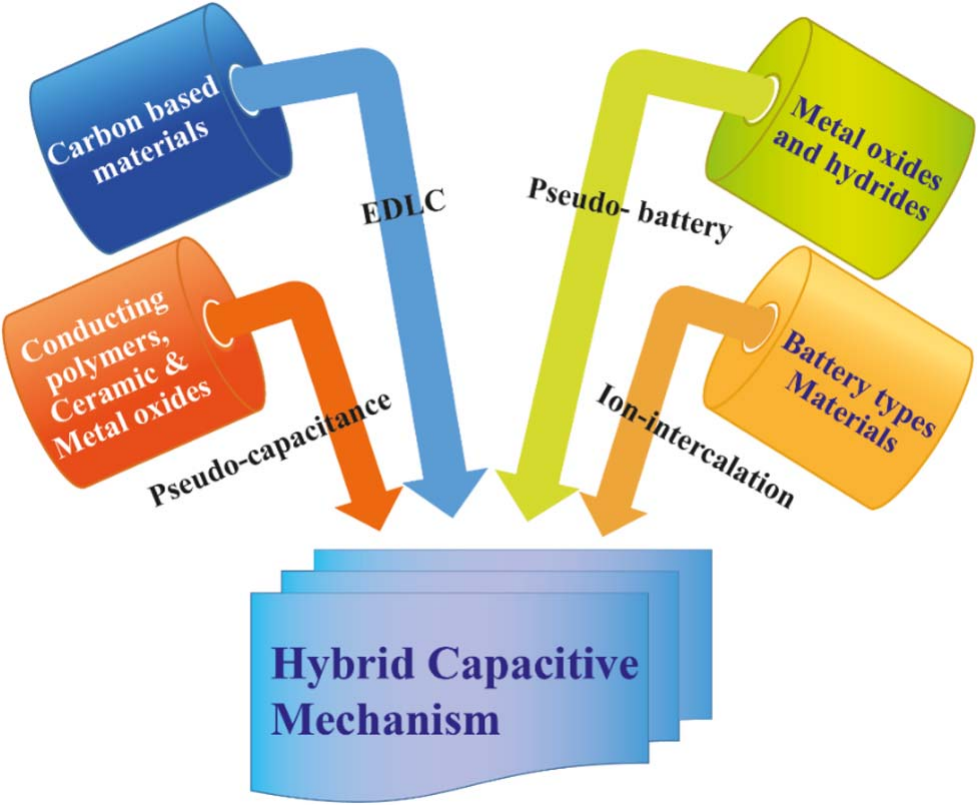
Schematic illustrations of different types of materials containing various types of charge storage mechanisms can merge to build up a hybrid capacitive mechanism *via* a controlled synthesis phenomenon.

By EDLC mechanism, researchers found that higher energy density can be achieved without altering power density and high cyclic stability. In the meantime, a battery-type metal ion-intercalation mechanism was also applied to super-capacitive behavior as a hybrid strategy for getting higher-performance devices. So, a new window is opened to touch our desired passion to achieve fast charging-discharging energy reservoir devices within higher capacitance value, high energy and power density, excellent rate capability along with cyclic stability and high potential windows by merging different types of materials that possessed different types of mechanism strategies shown below in Fig. 6. Finally, a lot of efforts and focus give on hybrid capacitive mechanism-based devices fabrication to achieve desired properties in modern era (Table 1).

**Table 1:** Summary of capacitive charge storage mechanisms, materials, properties

Mechanism	Materials/description	Property/characteristics	Electrochemical signature	Advantages	Disadvantages	Limitations	Examples	References
EDLC (electric double-layer capacitance)	Carbon-based materials	High conductivity, power density, non-faradaic electrostatic ion adsorption	CV: Rectangular, GCD: linear/triangular	Excellent power density, ultra-fast charge/discharge, very long cycle life, high stability & safety	Very low energy density	Limited to surface area, ineffective for long-term energy storage	Graphene, CNT, carbon fiber	33
Pseudo-capacitance (PC)	Conducting polymer, complex ceramics, metal oxides	High capacitance, cyclic stability, lightweight, flexibility, surface/near-surface reversible redox reactions	CV: Quasi-rectangular with small redox humps, GCD: Slightly nonlinear slope	Higher capacitance & energy density, faster response	Lower cycle stability, poor conductivity	Moderate stability, limited scalability	PANi, PPy, PEDOT, MnO_2_, RuO_2_	34
Pseudo-battery (PB)	Metal oxides and hydrides	Fast charging-discharging, long usability. Ion intercalation with partial diffusion control	CV: broad redox peaks (not sharp), GCD: nonlinear with plateau-like regions	Higher energy density than EDLC/PC, faster than batteries	Lower cycle life, slower kinetics	Mechanical stress, needs nanostructuring/carbon support	Ni(OH)_2_, NiO, Co_3_O_4_	13
Ion intercalation (battery-type)	Battery-type electrode materials	High energy density, higher capacity, bulk ion insertion/extraction with phase transition	CV: distinct sharp redox peaks, GCD: voltage plateaus	Very high energy density, long-duration storage	Poor power density, mechanical degradation	Significant volume change, poor long-term cycling	LiCoO_2_, LiTi_4_O, LiFePO_4_	35

## 4. Fabrication and architectures of hybrid capacitive nanocomposites

### 4.1 Design and fabrication strategies of electrode materials

The performance of supercapacitors is deeply connected to the way electrode materials are created and structured. Various synthesis methods are available to adjust physical form, pore structure, electrical conductivity, and flexibility, all of which help enhance energy storage capability. A comparative overview of flexible electrode fabrication methods has been shown in Table 2. Hydrothermal synthesis: these methods use solutions to produce well-defined nanocrystals with specific shapes like nanorods, nanosheets, or hollow spheres. These structures improve the movement of ions and electrons and increase surface availability.^36^*In situ* polymerization: conducting polymers such as PANi, PPy, and PEDOT can be grown directly onto conductive backings. This ensures excellent contact at the interface, even coating, and the flexibility needed for bendable and wearable electronics.^37^

**Table 2:** Comparative overview of flexible electrode fabrication methods

Fabrication method	Typical structure	Electrochemical performance	Advantages for flexible/wearable devices	References
Hydrothermal/solvothermal	Nanorods, nanosheets, hollow spheres	High surface area → enhanced ion/electron transport; good capacitance	Controlled morphology; scalable; tunable crystallinity	36
*In situ* polymerization	Conducting polymer coatings on substrates (PANi, PPy, PEDOT)	High capacitance; good cycling stability	Strong interfacial contact; uniform coating; mechanical flexibility; suitable for wearable electronics	37
Sol–gel processing	Homogeneous oxide networks	Tunable porosity and crystallinity; high capacitance; long-term cycling stability	Molecular-level precursor mixing; easily tailored porosity; compatible with flexible substrates	38
Electrospinning	1D nanofibers; interconnected porous networks	High surface area; enhanced conductivity; good charge storage	Excellent mechanical flexibility; supports symmetric and hybrid devices; lightweight	39
Electrodeposition	Conformal coatings/thin films	Tunable thickness; strong adhesion; improved cycling stability	Direct deposition on current collectors; uniform films; adaptable for flexible substrates	40 and 41
Templating/biomass-derived carbons	Hierarchical porous structures	Competitive capacitance; good rate capability	Low cost; sustainable; hierarchical porosity enhances ion transport; adaptable for flexible devices	42 and 43

Sol–gel processing: the sol–gel technique allows precursors to mix at the molecular level, resulting in uniform oxide networks with adjustable porosity and crystal structure. This method can lead to high capacitance and stable performance over many charge–discharge cycles.^38^

Electrospinning: this flexible method creates one-dimensional nanofibers that form interconnected porous networks. Carbon-based materials and composites made this way offer high surface area and good conductivity, making them suitable for both symmetric and hybrid devices.^39^ Electrodeposition: this is a controllable way to deposit uniform coatings and thin films directly onto current collectors. Electrodes made this way have adjustable thickness, adhere strongly to the substrate, and often show improved long-term cycling stability.^40,41^ Templating and biomass-derived methods: using hard or soft templates, as well as carbons produced from sustainable biological waste, can create materials with multiple levels of porosity and tunable surface properties. These approaches provide competitive capacitance in a cost-effective manner.^42,43^ In summary, these fabrication techniques allow scientists to build zero-dimensional nanoparticles, one-dimensional nanofibers or nanotubes, two-dimensional nanosheets, and three-dimensional porous frameworks. Each structure offers specific advantages for ion and electron transport. A solid grasp of these methods is key to developing high-performance supercapacitor technologies that can be produced on a larger scale.

### 4.2 Nanocomposite designs for hybrid capacitive storage

**Fig. 7:**
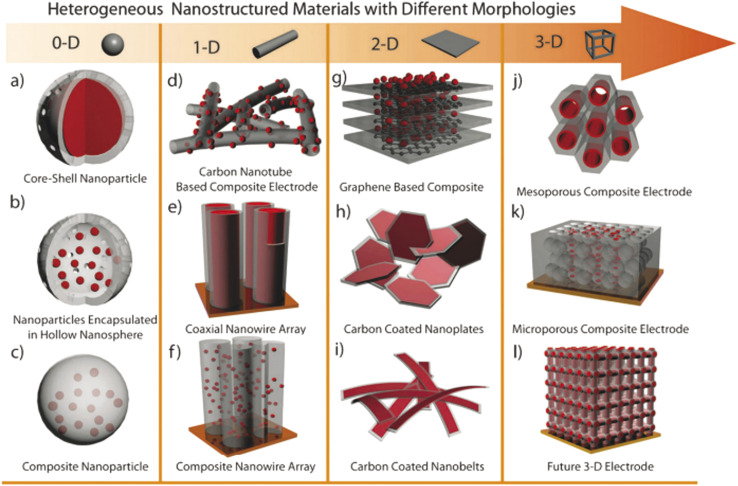
Schematic of heterogeneous nanostructures based on (a–c) 0D, (d–f) 1D, (g–i) 2D and (j–l) 3D nanostructures.^46^ [Reproduced from ref. 46 with permission from The Royal Society of Chemistry, copyright 2025].

Nano-structuring enhances the higher surface area, excellent carrier mobility and electrochemical activity of energy reservoir materials, building up superior performances in the energy harvesting field. Particularly, several nanostructured configurations are invented, including nanofibers, hollow spheres, nano-rods, nano-bowls, nanotubes and ultrathin films.^44^ Vertically aligned nanowire arrays have emerged as one of the most promising nanostructured architectures for energy storage applications, offering distinct advantages over randomly oriented nanowires. Firstly, each nanowire is directly and electrically connected to the underlying conductive substrate, ensuring that the entire array contributes to the overall capacity. Secondly, the vertically aligned configuration provides direct one-dimensional (1D) electron pathways, facilitating efficient charge transport and reducing ion diffusion distances, which collectively enhance high-rate electrochemical performance. Thirdly, in contrast to bulk or micron-scale materials, the inter-nanowire spacing in these arrays can effectively accommodate volume changes during repeated charge/discharge cycles, thereby mitigating mechanical degradation and improving structural stability.^45^ Based on capacitive mechanisms, materials are divided into different category where every types of materials processing different kinds of energy reservoir performances (Fig. 7). Information on EDLC and carbon materials have been provided more detailed in Section 2.1 and in Table 3.^1,47^

**Table 3:** Nano-structured materials with morphology and electro-chemical performance for energy storage devices (*i.e.* carbon materials, conducting polymer, complex structured ceramic)

Structural morphology	Electrode materials	Chemical structure	Property	TEM/SEM image	Electrolyte	Specific capacitance (F g^−1^)	Ref.
Carbon-based 0D nano-sphere	Fullerene (C-60)	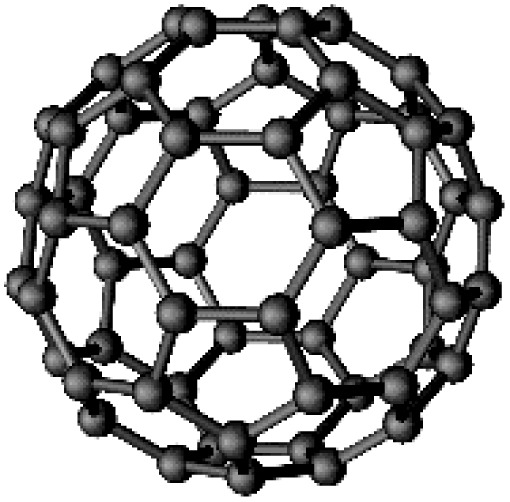	Perfect electron acceptor	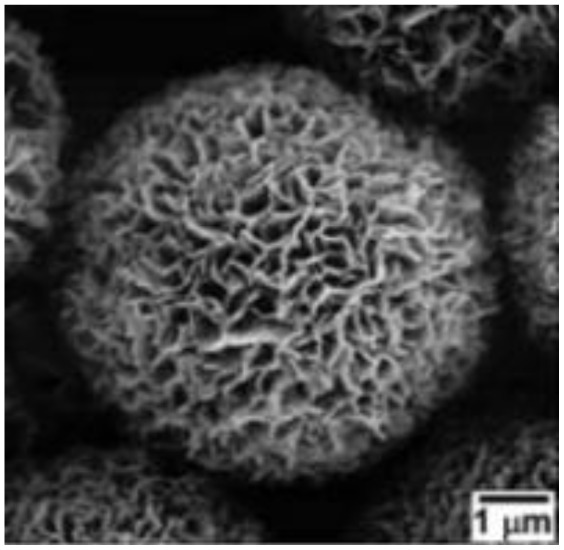	6 M KOH	505.4	54
0D nano-particle	Carbon black	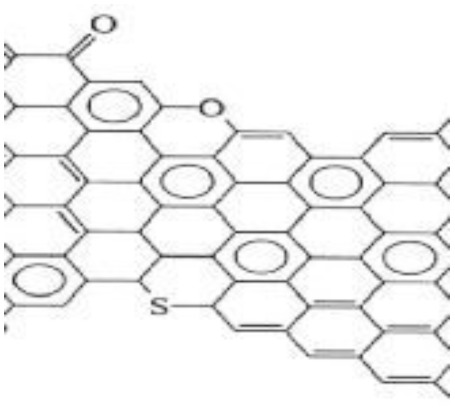	Para-crystalline carbon, act as a spacer	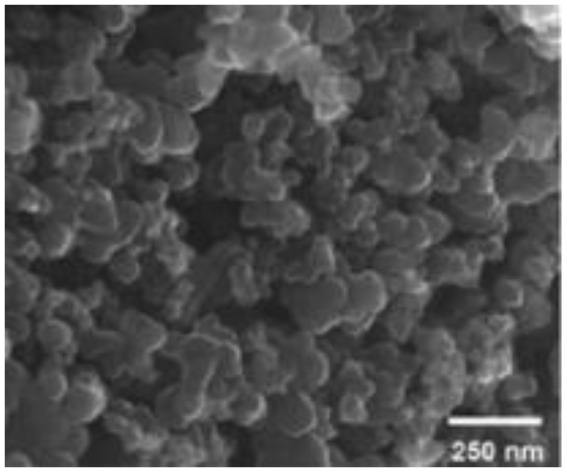	Aqueous (NaOH/KOH)	<300	15 and 55
1D nano-tube	Carbon nano-tube (CNT)	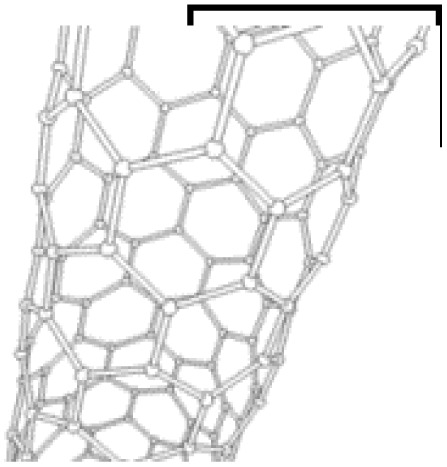	High aspect ratio, good mechanical property	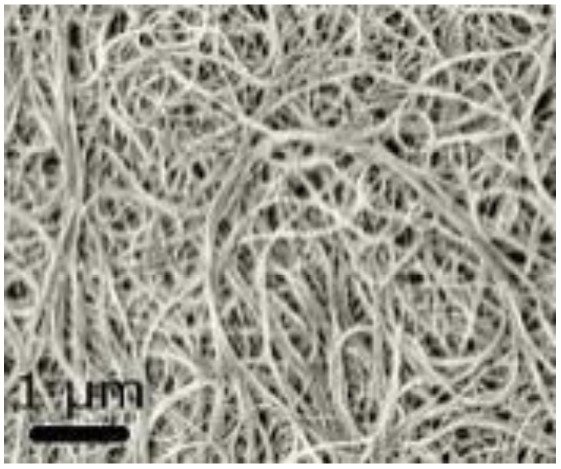	Aqueous (NaOH/KOH)	20–180	15
2D nano-sheet	Graphene	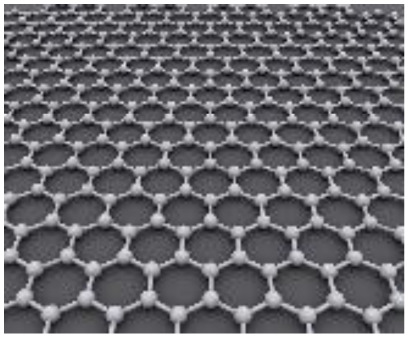	High surface area, good electrical conductivity	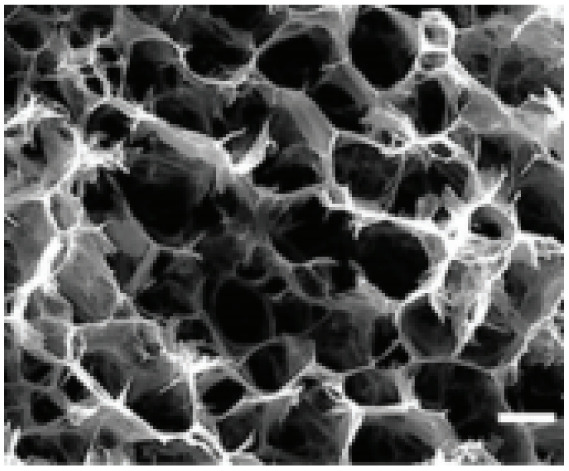	5.5 M KOH	135	56 and 57
2D nano-sheet	Reduced graphene oxide	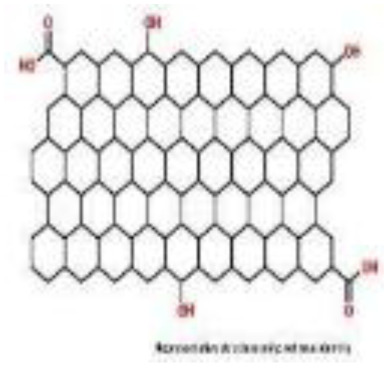	Easy process ability, defect healing system	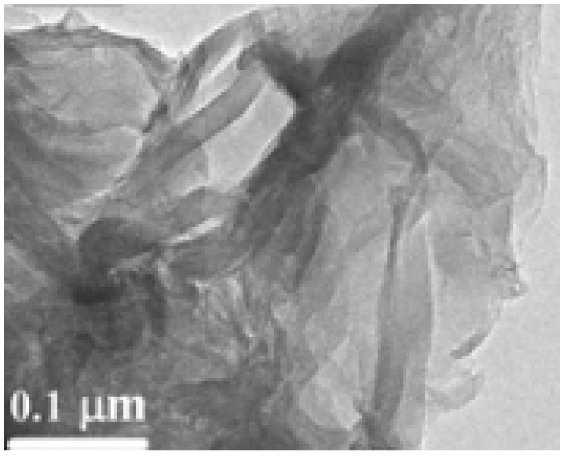	1.1 M Fe(CN)_6_	223.6	58
3D nano-intrinsic porous	Activated carbon	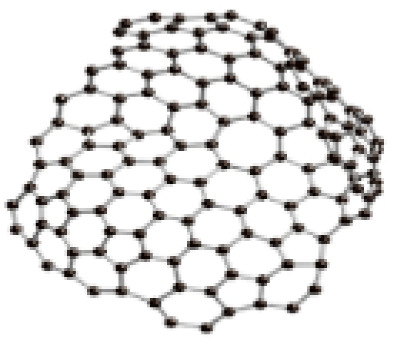	High packing density	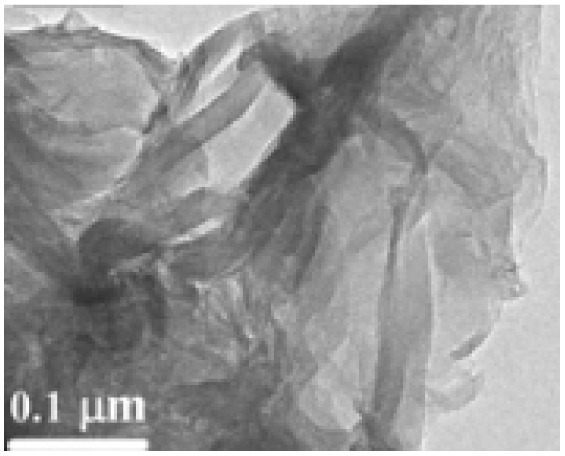	Aqueous (NaOH/KOH)	200–400	15 and 59
3D connected nanoparticle	Carbon aerogels	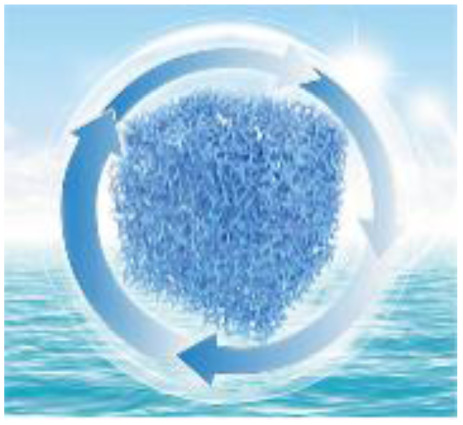	Bimodal pore structure	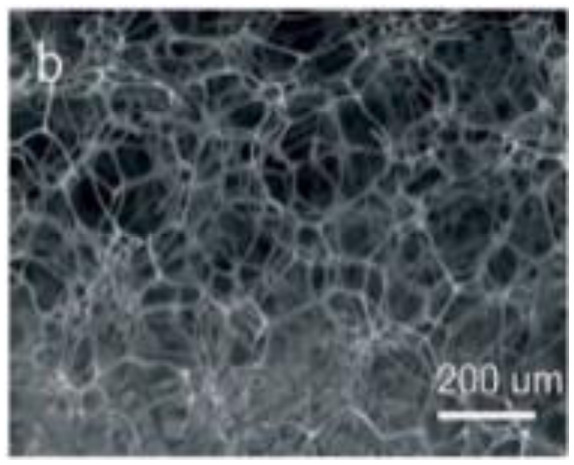	Aqueous (NaOH/KOH)	40–200	15 and 60
Conducting polymer, 0D nano-particle	Polyaniline (PANI)	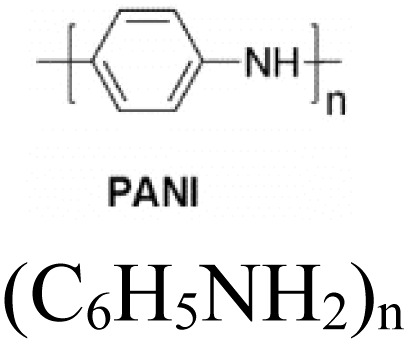	30–200 (S cm^−1^)	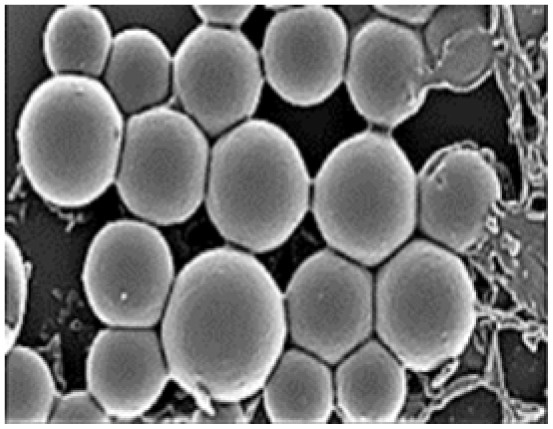	H_2_SO_4_ aqueous	408	61 and 62
0D nano sphere	Polyaniline (PANI)	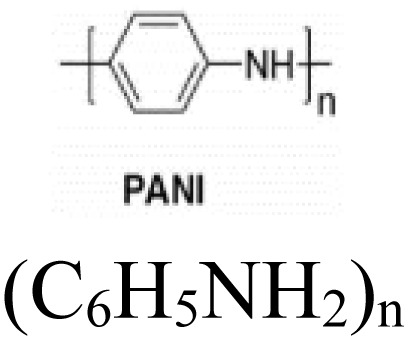	30–200 (S cm^−1^)	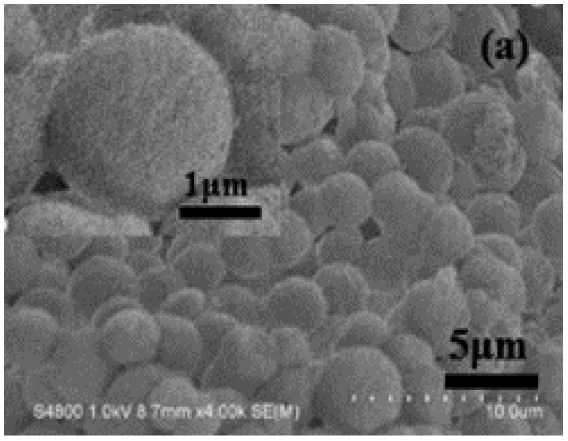	H_2_SO_4_ aqueous	421	44 and 63
1D nanowire arrays	Polyaniline (PANI)	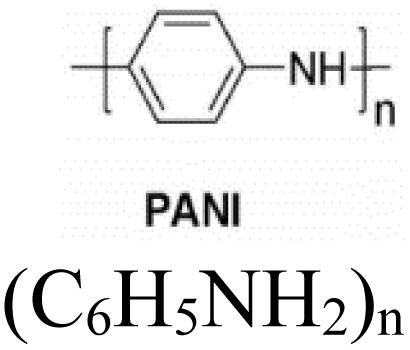	30–200 (S cm^−1^)	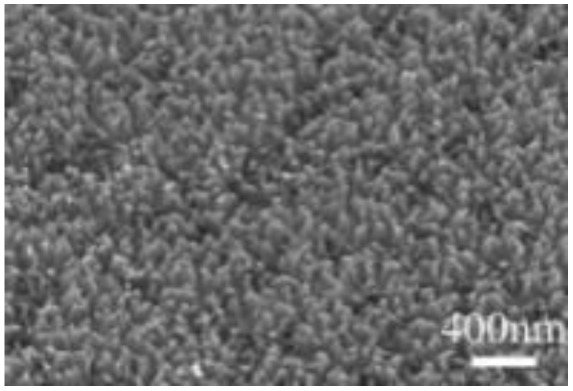	HClO_4_ aqueous	950	64
1D nano-tube	Polyaniline (PANI)	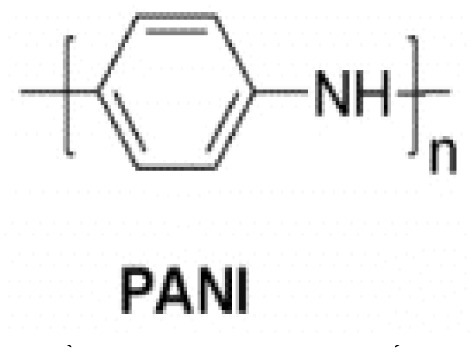	30–200 (S cm^−1^)	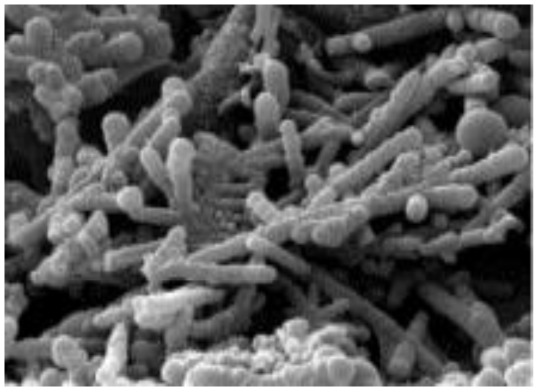	HCl aqueous	522	64 and 65
0D nano-particle	PTh	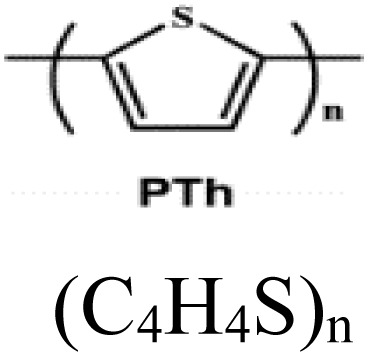	10–1000 (S cm^−1^)	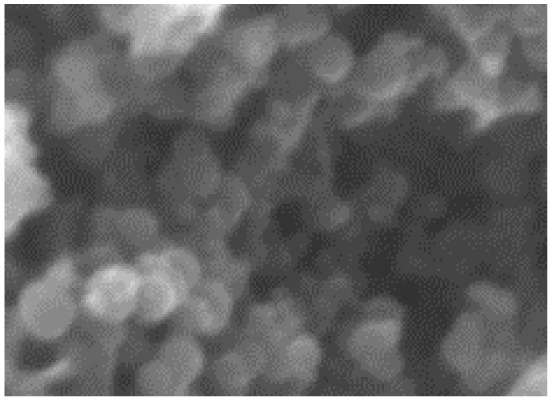	Polymer electrolyte membrane	156	33
1D nanofibers	PEDOT	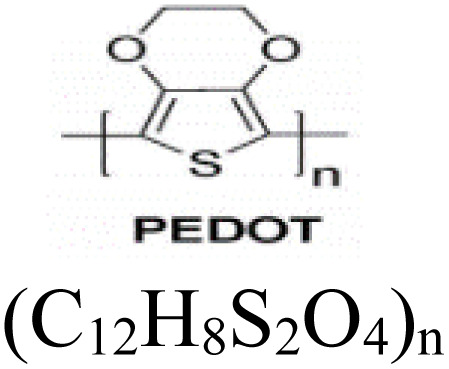	0.4–400 (S cm^−1^)	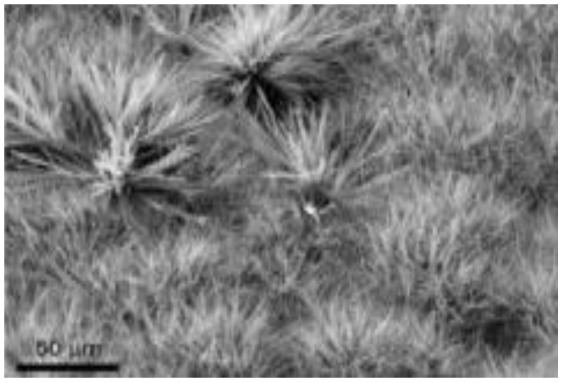	6 M HCl	175	44 and 66
3D structure connect by nano-sphere	Polypyrrole (PPy)	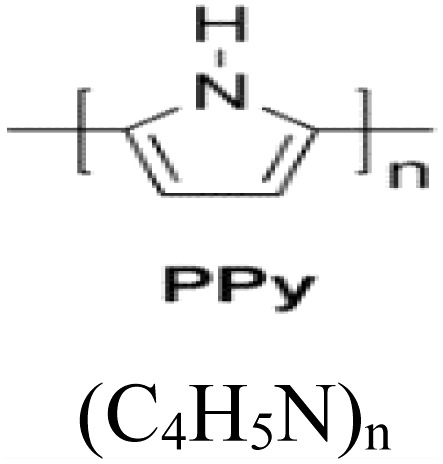	10–7500 (S cm^−1^)	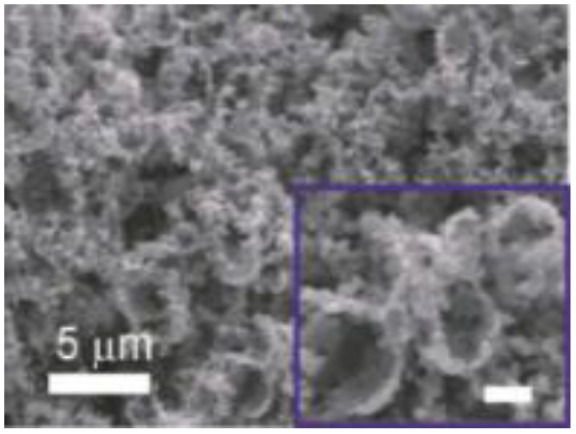	PVA–H_2_SO_4_	132	44 and 67
Complex structured ceramic, 0D nano-particle	Spinel (AB_2_O_4_) CoFe_2_O_4_	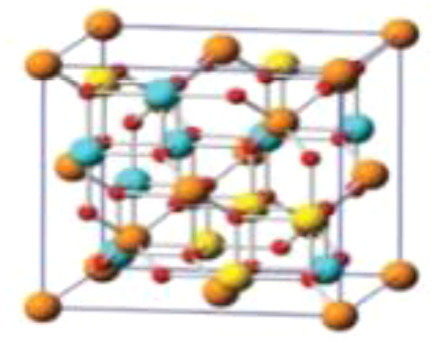	Excellent chemical stability, high capacity	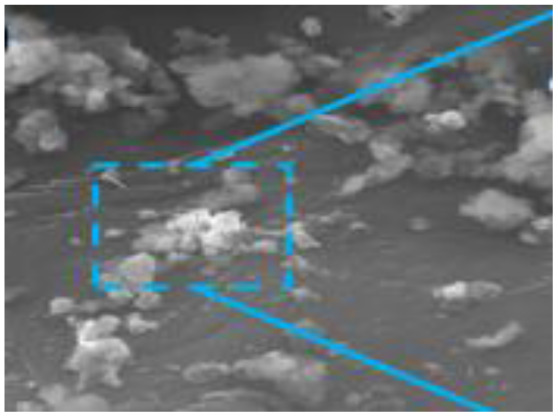	1 M KOH	123	52 and 68
1D nanowire	NiCo_2_O_4_	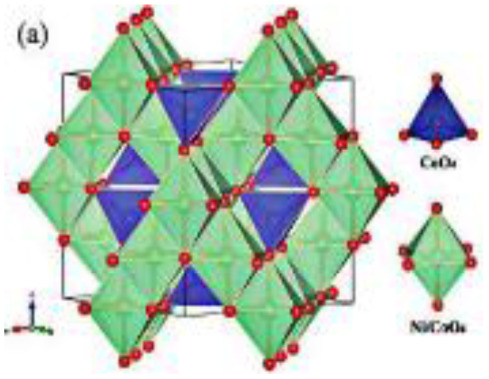	Highly crystalline nano particles	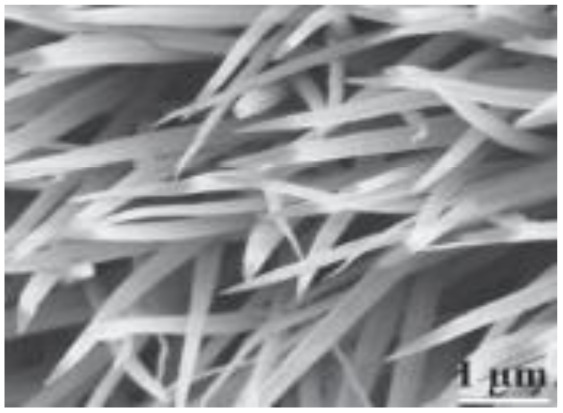	6 M KOH	1283	19
2D nano-sheets	NiCo_2_O_4_	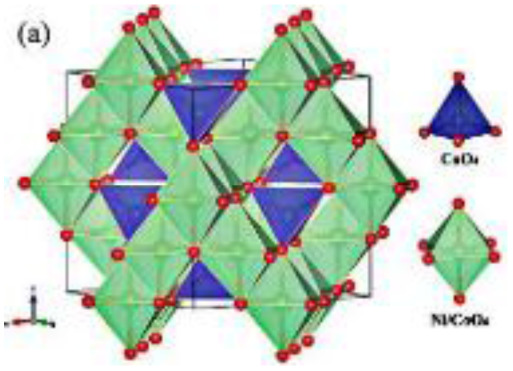	Fast electron & ion transport, structural stability	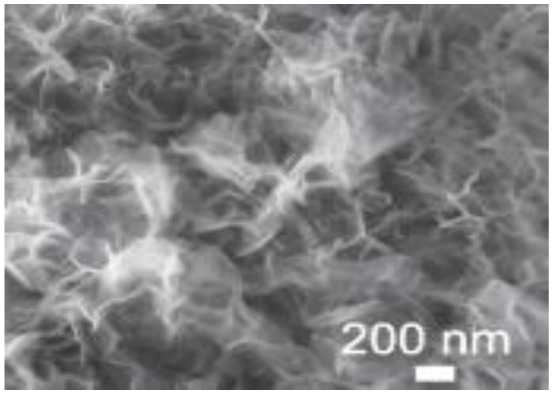	3 M KOH	2010	69
3D flower like	NiCo_2_O_4_	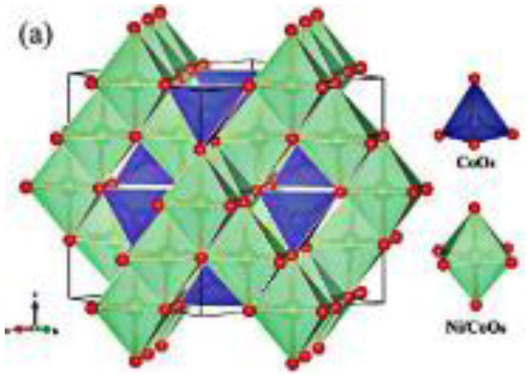	Ions diffusion- pathway	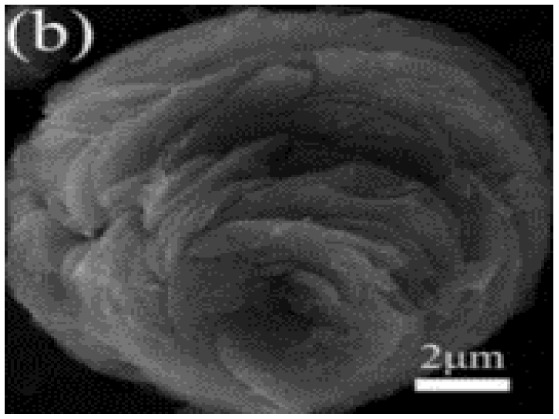	6 M KOH	658	57 and 69
3D nano-cube	MnFe_2_O_4_	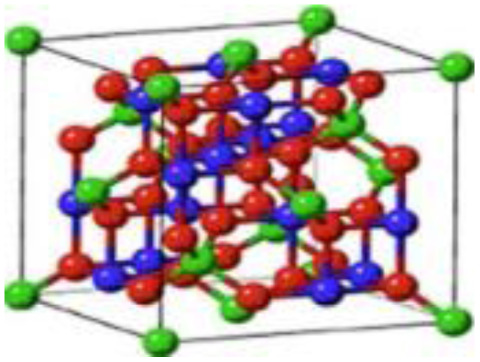	Higher surface area, high active sites	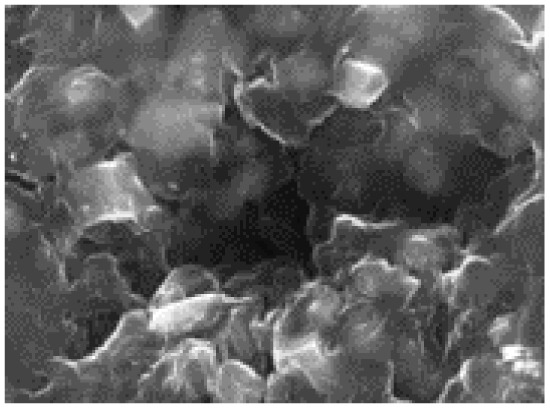	1 M NaCl	45	70 and 52
0D nano-particle	Perovskite (ABO_3_), LaMnO_3_	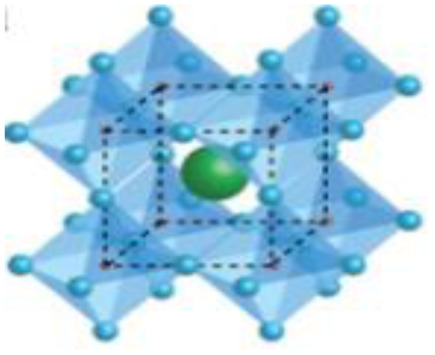	Significant electrochemical activity	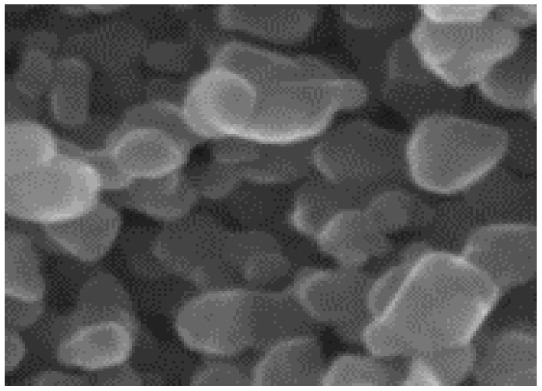	0.5 M Na_2_SO_4_	520	22 and 71
0D nano particle	SrRuO_3_	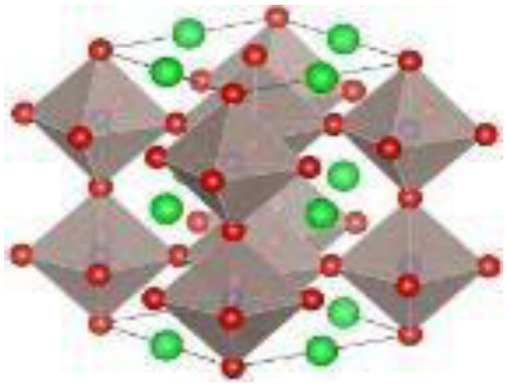	Enhances electro chemical performance	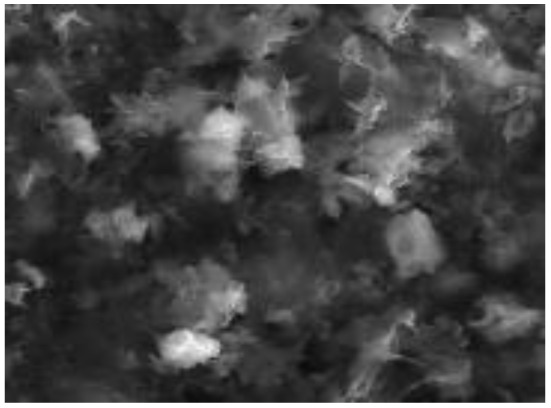	1 M KOH	52.4	72
1D nano-tube	LaFeO_3_	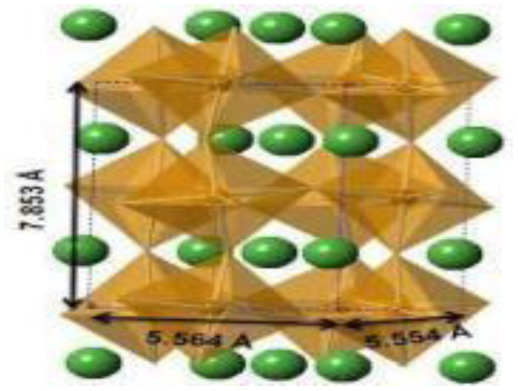	Large surface area, small resistance	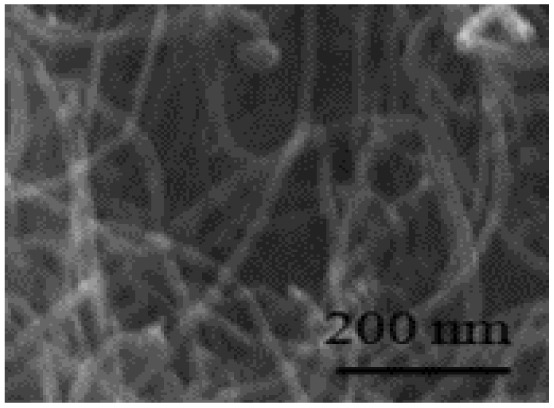	2 M KOH	313.21	73
2D nano-sheet	LaNiO_3_	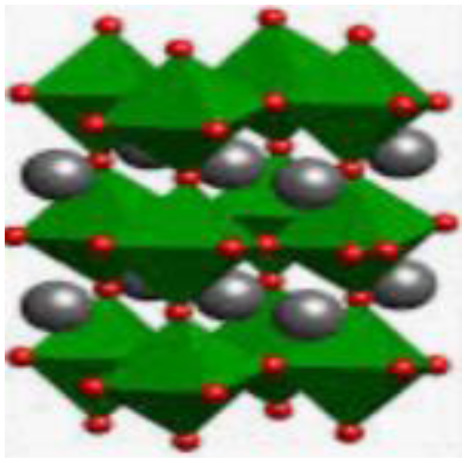	High conductivity, rich porous-structure	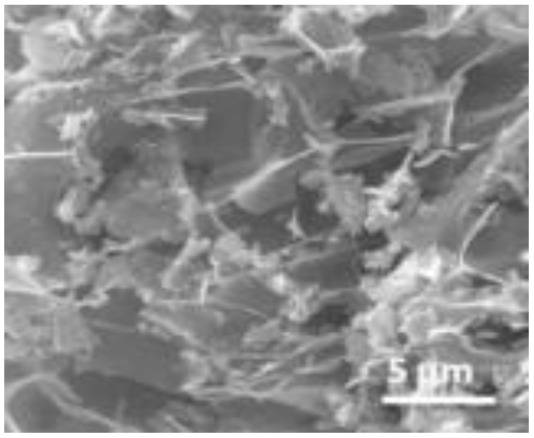	6 M KOH	139.2	74
3D nano-flakes	BiFeO_3_	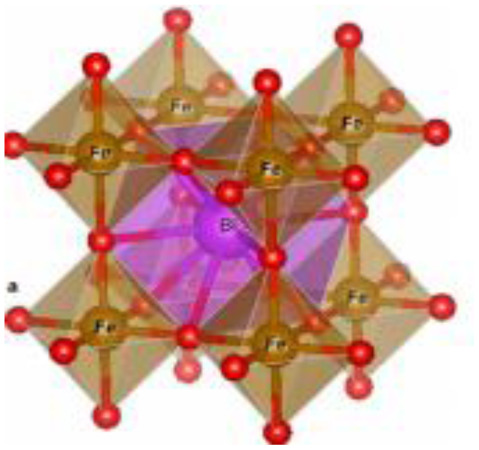	High temperature stability	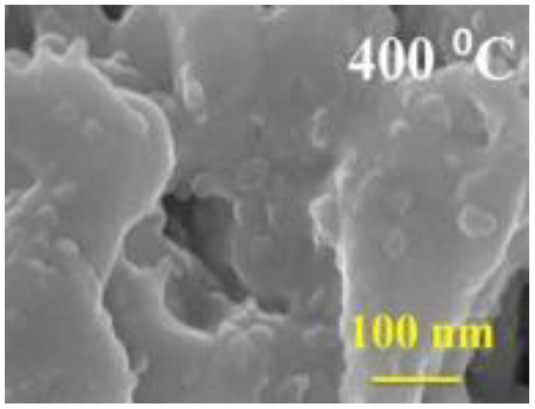	2 M NaOH	72	75

**Table 4:** Nano-structured carbon-based, CP and ceramic materials within binary and ternary composite for flexible energy storage devices and electro-chemical performance

Structural morphology	Electrode materials	Synthesis method	Electrolyte	Specific capacitance (F g^−1^)	Capacity retention	Power density (Wh kg^−1^)	Ref.
0D binary composite	PANI/carbon particle	*In situ* polymerization	H_2_SO_4_/PVA gel	272.6	95.7% after 501 cycles	—	93
	Nanoparticle PANI/graphene	—	—	257	98% after 1000 cycles	—	64
1D binary composite	PANI/CNT	*In situ* electrochemical polymerization	1 M non-aqueous LiClO_4_	236	∼85% after 1000 cycles	131	77
	PANI/carbon nanofiber	Electrochemical polymerization	1 M H_2_SO_4_	366	80% after 1000 cycles	—	94
	PPy/CNT	*In situ* chemical polymerization	7.5 M KOH	265	—	—	95
	PANI/MWCNT	—	0.1 M H_2_SO_4_	560	—	—	96
	PANI/MWCNT	—	1 M NaNO_3_	328	94% after 1000 cycles	—	97
	PTh/MWCNT	Electrochemical polymerization	0.5 M H_2_SO_4_	110	90% after 1000 cycles	—	91
	PANI/SWCNT	—	1 M H_2_SO_4_	485	94% after 1500 cycles	—	8
2D binary composite	PANI/graphene	*In situ* polymerization	2 M H_2_SO_4_	480	>70% after 1000 cycles	—	98
	Reduced GO	*In situ* polymerization	1 M H_2_SO_4_	701	92% after 1000 cycles	—	99
	PPy/GO	*In situ* surface-initialed polymerization	1 M H_2_SO_4_	370	91.2% after 4000 cycles	—	77
	PEDOT/rGO	*In situ* polymerization	—	108	—	—	100
	PEDOT/GO	*In situ* polymerization	1 M H_2_SO_4_	270	—	—	101
	PEDOT/rGO	Polymerization	1 M H_2_SO_4_	213	—	—	90
	PEDOT/GO	Electrochemical deposition	0.5 M H_2_SO_4_	715	—	—	102
	Nano-Cone PANI/Graphene	Electro-deposition	1 M HClO_4_	750	—	—	81
	Nano-tube PANI graphene	Chemical oxidative polymerization	—	—	91.4% after 500 cycles	74.27	103
	Nano-wire PANI graphene	Chemical polymerization	—	740	87% after 1000 cycles	—	104
	PPy graphene	*In situ* polymerization	3 M KCl	255.7	> 93% after 1000 cycles	7.02 Wh kg^−1^	77
3D binary composite	Nano-wire PANI/carbon cloth	—	1 mol L^−1^ H_2_SO_4_	1079	14% loss after 2100 cycles	—	105
	Nano-wire PEDOT/carbon cloth	—	1 mol L^−1^ Na_2_SO_4_	256	30% loss after 1000 cycles	—	106
	PEDOT/multilayer graphene	Electrochemical deposition	1 M H_2_SO_4_	154	—	—	107
0D ternary composite	Nanoparticle CoFe_2_O_4_/(PANI)/(rGO)	*In situ* chemical oxidative polymerization	1 M KOH	9	—	270 × 10^−8^ Wh cm^−1^	52
	Nanospheres NiCo_2_O_4_/Ppy/carbon textiles	Chemical polymerization	PVA/KOH gel	2244	—	58.8 Wh kg^−1^ at 365 W kg^−1^	78
1D ternary composite	NiCo_2_O_4_/polypyrrole nanowires	Hydrothermal	3 M NaOH	2055	—	—	104 and 108
	Nano composite BaTiO_3_/(PPy)/MWCNT	*In situ* oxidative polymerization	—	155	—	21.6 Wh kg^−1^ at 385.7 W kg^−1^	108
2D ternary composite	Nanocomposite LaMnO_3_/PANI/RGO	*In situ* polymerization	1 M HCl solution	111	—	50 Wh kg^−1^ at 2.25 kW kg^−1^	82
	Nano-sheet SrTiO_3_/Ppy/modified graphene	*In situ* oxidative polymerization	6 M KOH	466	—	165.69 Wh kg^−1^ at 5598 W kg^−1^	109
3D ternary composite	Nano-cube MnFe_2_O_4_/(PANI)/graphene	*In situ* chemical oxidative polymerization	1 M NaCl aqueous	338	—	10.25 Wh kg^−1^ at 3076 W kg^−1^	70
	Mesoporous nano-sheets NiCo_2_O_4_ Ni foam	Co-electro deposition	3 M KOH aqueous	1450	—	—	72

Reducing materials to the nanoscale significantly alters their physical and chemical properties, often resulting in enhanced performance for electrochemical applications. In particular, nanomaterials are expected to play a crucial role in advancing energy storage devices due to their increased electrode–electrolyte interfacial area and shortened ion/electron transport pathways, both of which improve charge storage and transfer kinetics. The hybridization of conducting polymers with carbon nanomaterials has shown promising potential by combining the redox activity of conducting polymers with the exceptional electrical conductivity and mechanical robustness of carbon-based materials, leading to superior electrochemical performance. Conducting polymers offer several advantages for supercapacitor applications, including low cost, environmental compatibility, high electrical conductivity in the doped state, broad electrochemical voltage windows, large specific capacitance, high porosity, excellent reversibility, and tunable redox properties through chemical modification.

In these systems, charge storage occurs *via* faradaic redox reactions throughout the bulk of the conducting polymer. Upon oxidation, cations from the electrolyte are incorporated into the polymer backbone, while during reduction, these ions are released back into the electrolyte. Unlike battery-type electrodes, this redox process does not involve structural phase transitions, thus ensuring high reversibility and cycling stability. Notable examples of intrinsically conducting polymers include polypyrrole (PPy), polyaniline (PANI), polythiophene (PTh) and its derivatives, poly(3,4-ethylenedioxythiophene) (PEDOT), and poly(*p*-phenylenevinylene) (PPV) and related compounds.^48^

However, while binary composites of polymers and carbon nanomaterials offer improved performance, their energy densities still lag behind those of batteries. To address this limitation, researchers have developed ternary composites integrating complex ceramic materials, conducting polymers, and carbon nanostructures. These systems demonstrate synergistic effects, delivering enhanced energy density without compromising power density, along with excellent cycling stability, high specific capacitance, low internal and diffusive resistance, and large electrochemically active surface areas. Such properties make them ideal candidates for flexible and wearable energy storage devices suited to modern electronics.

In the current study, emphasis is placed on ternary composites involving complex structured ceramics, conducting polymers, and carbon materials for the fabrication of flexible hybrid supercapacitors. Among the ceramic components, inverted or partially inverted spinel-structured compounds have garnered significant interest due to the presence of multi-valent cations at both tetrahedral and octahedral sites, which enhance charge storage capability. Miao *et al.* demonstrated a NiFe Prussian blue analogue/reduced graphene oxide composite cathode for aqueous sodium-ion hybrid supercapacitors, achieving enhanced ion transport, high capacitance, and stable cycling performance.^49^ Their work highlights the role of composite electrode design in advancing next-generation hybrid supercapacitors. Gupta *et al.* developed an Fe-based Prussian blue analogue anchored on reduced graphene oxide to suppress metal dissolution and enhance conductivity, thereby improving cycling stability and rate performance in aqueous sodium-ion supercapatteries.^50^ This work underscores the importance of hybrid electrode engineering for durable and high-power energy storage devices. Kankane *et al.* incorporated halloysite nanotubes into electrospun PVDF-HFP separators, achieving improved ionic conductivity and interfacial stability in sodium-ion batteries, highlighting the role of nanostructured polymer composites in enhancing device performance.^51^ Li *et al.* designed cellulose nanofiber–silver nanowire/tungsten trioxide hybrid films as paper-based transparent electrochromic supercapacitors, demonstrating high stability and bifunctionality, relevant for flexible and multifunctional energy storage applications.

Conducting polymers are selected for their high theoretical capacity, chemical stability, and electrocatalytic efficiency, making them indispensable components in next-generation energy storage systems.^52^ As every material has an individual specific mechanism to contribute to increasing capacitive property, the ternary hybrid materials may result excellent and perfect device electrode to use in practical applications where a huge energy storage system is required in a short period. Mainly, hybrid energy storage devices are run by four mechanisms, which were discussed broadly in earlier sections. But nowadays, a new complex structured ceramic material is used as conducting and capacitive materials as it possesses high dielectric property, ferroelectricity, piezo-electricity, pyro-electricity *etc.*, which is also required pseudo-capacitive mechanism.^53^ But it has a limitation, that is, possessing low electrical conductivity, though it has high pseudo-capacitive property. Hence, researchers have drawn attention to combining these new materials with conducting polymer (high capacitive property, flexibility, reversible redox reaction *etc.*) and carbon materials (high conductivity, mechanical support, EDL capacitive property *etc.*) for exploring excellent and perfect device electrode materials.

Recently, nano-structure-based complex structured ceramics are commonly used as a new highly capacitive material. Several ceramic materials having complex crystal structures are used in electrical device applications that are electrically and magnetically conductive due to their internal structure phenomena. There are several types of structure, *i.e.*, spinel, perovskite, silicates, silica, olivine, garnets, ring silicates, micas, clay minerals, pyroxene, b-aluminas, calcium aluminate, mullite, monazite, YBa_2_Cu_3_O_7_, Si_3_N_4_.^54^ Among these complex structures, ceramic, spinel and perovskite are more popular and advantageous in energy storage device applications as all of them possess ferromagnetic properties, high dielectric properties due to internal structure mechanism (Table 3). Spinel has the general formula AB_2_O_4_, although later we also write it as AO. *n*B_2_O_3_, where *n* describes the non-equi-molarity.^53^ A great variety of compositions can possess this complex crystal structure due to its intrinsic stacking system in the crystal pattern. They are MgAl_2_O_4_, NiFe_2_O_4_, NiCo_2_O_4_, MnFe_2_O_4_, CoFe_2_O_4_*etc.*, which holda spinel crystal structure. Nanocomposite materials have transformed hybrid energy storage by leveraging the synergistic integration of carbonaceous, polymeric, and ceramic components, resulting in increased conductivity, structural stability, and multifunctional electrode designs optimized for high-performance supercapacitive systems.

## 5. Nanocomposite materials

### 5.1 Zero-dimension

Zero-dimensional (0D) or dimensionless materials refer to particles with a nearly spherical morphology, possessing an aspect ratio close to 1. Examples of 0D materials include fullerenes, quantum dots, nano-onions, and nanoparticles, which exhibit roughly spherical shapes.^76^ A key advantage of these materials lies in their tunable pore content and size distribution, making them highly suitable as support materials in supercapacitor electrodes.

Various polyaniline (PANI)–carbon nanocomposites have been explored, including PANI/carbon spheres and PANI/carbon particles.^77^ For instance, Shen *et al.*^78^ studied the electrochemical performance of a Nano-PANI/hollow carbon sphere composite synthesized *via* an *in situ* polymerization method. Electrochemical testing revealed that the composite achieved a high specific capacitance of 435 F g^−1^ at a current density of 0.5 A g^−1^ and maintained 60% of its initial capacitance after 2000 cycles. In another study, Vijaya Sankar *et al.*^52^ developed a novel nanocomposite consisting of CoFe_2_O_4_ nanoparticles, reduced graphene oxide (rGO), and PANI using an *in situ* chemical oxidative polymerization method. The capacitance properties were optimized by tuning the component ratios and material architecture, achieving a specific capacitance of approximately 8.59 F m^−1^ at a scan rate of 1 mV s^−1^ in a 1 M KOH electrolyte.

**Fig. 8:**
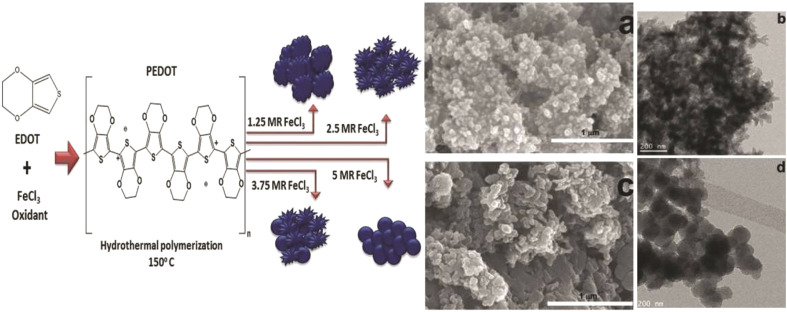
Schematic representation of the formation of various crystalline PEDOT nano-dendrites and nano-spheres by hydrothermal polymerization with SEM images of PEDOT nano-particles obtained from the oxidant (a) 1.25 MR of FeCl_3_ (c) 2.5 MR of FeCl_3_ with TEM images of PEDOT nanoparticles prepared using (b) 3.75 MR of FeCl_3_ and (d) 5 MR of FeCl_3_.^79^ [Reproduced from ref. 79 with permission from The Royal Society of Chemistry, copyright 2025].

Murugesan Rajesh and his group *et al.* introduce poly (3, 4-ethylenedioxythiophene) (PEDOT) by a hydrothermal polymerization process using various types of FeCl_3_ and resulting in good conductive, crystalline PEDOT nano-dendrites and nano-spheres. In summary, it is a promising way to synthesize carbon materials/CP/CM composites to improve the electrochemical performance of SC (Fig. 8).^79^

### 5.2 One-dimension

One-dimensional (1D) nanostructures are fiber-shaped materials characterized by their high aspect ratio.^80^ Their unique dimensionality significantly influences their functional properties, making them attractive for energy storage applications. Typical 1D nanostructures include nanotubes, nanofibers, nanowires, nanopillars, nanoribbons, and nanobelts.^81^ Examples such as carbon nanotubes (CNTs), conducting polymer (CP) nanowire arrays, and carbon-based nanofibers (CM nanofibers) are prominent 1D carbon nanostructures. These materials exhibit excellent electronic transport properties and high aspect ratios, which enhance the kinetics of electrochemical reactions.^76^

**Fig. 9:**
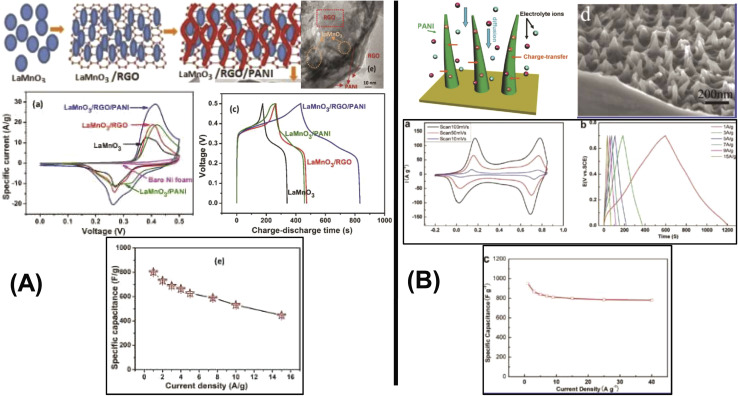
Comparison between two papers where one shows schematic illustration of the formation of LaMnO_3_/RGO/PANI including 0D Perovskite structured LaMnO_3_ nano-particles (A) and schematic of the optimized ion diffusion path in PANI nanowire arrays in HClO_4_ aqueous solution (B) and also comparing with the HRTEM and SEM images, CV curves at different compositions and scan rates, galvano-static charge discharge curves at different compositions and cycle stability with capacitance retention value within multi-dimensional devices.^82,86^ [Reproduced from ref. 82 and 86 with permission from The American Chemical Society, copyright 2025].

Recent advancements have demonstrated the potential of 1D nanostructures in hybrid composites. P. Muhammed Shafi *et al.* synthesized a LaMnO_3_/RGO/PANI composite *via* a two-step *in situ* polymerization method.^82^ The integration of reduced graphene oxide (RGO) and PANI with LaMnO_3_ nanoparticles improved structural stability, electrical conductivity, and electrochemical performance. Imani and Farzi fabricated a PANI/multi-walled carbon nanotube (MWCNT) nanocomposite with a tubular morphology using a low-temperature *in situ* polymerization method.^83^ When the MWCNT content reached 10%, the composite achieved a specific capacitance of 552.11 F g^−1^ at 4 mA cm^−2^, outperforming pure PANI (411.52 F g^−1^). The authors highlighted the potential of this low-temperature method for large-scale synthesis of tubular PANI/MWCNT structures. Niu *et al.* introduced a “skeleton/skin” strategy for preparing flexible, free-standing PANI/single-wall CNT (SWCNT) composite films using *in situ* electrochemical polymerization.^84^ In this configuration, the SWCNTs formed a continuous reticulate “skeleton” while PANI served as the conductive “skin.” The composite achieved a high specific capacitance of 236 F g^−1^ with a 30 seconds PANI deposition time, significantly surpassing that of pure SWCNTs (23.5 F g^−1^). Another noteworthy example involves carbon nanofibers. Birkl *et al.* reported the fabrication of 3D freestanding supercapacitor electrodes composed of PANI and porous carbon nanofibers. Compared to pure carbon nanofiber electrodes, the hybrid system exhibited a superior specific capacitance of 366 F g^−1^ at 100 mV s^−1^, attributed to the pseudocapacitive properties of PANI (Fig. 9).^82,85,86^

### 5.3 Two-dimension

Two-dimensional (2D) nanostructures are characterized by their sheet-like morphology and high aspect ratio. Notable examples of 2D carbon-based nanomaterials include graphene, graphene oxide (GO), and reduced graphene oxide (rGO).^76^ Generally, materials classified as 2D possess atomic-scale thickness, while their lateral dimensions extend well beyond the nanoscale range.^81^

Du and colleagues synthesized polyaniline (PANI)/graphene nanosheet (GNs) composites under varying conditions and assessed their electrical conductivity.^24,87^ Their findings revealed that increasing the GNs content enhanced the conductivity of the composites. This improvement was attributed to strong π–π interactions between PANI and GNs, where the GNs served as a structural template, promoting more planar and ordered PANI chains. Graphene oxide, a widely studied derivative of graphene, has been integrated into many conducting polymer (CP) composites. For instance, Wang *et al.* developed a high-performance electrode material by doping fibrillar PANI with GO *via* a soft chemical synthesis route.^88^ The resulting nanocomposite demonstrated excellent conductivity (10 S cm^−1^ at 22 °C) and a significantly enhanced specific capacitance of 531 F g^−1^ within a potential window of 0 to 0.45 V at a current density of 0.2 A g^−1^. This was markedly higher than that of pristine PANI (216 F g^−1^), highlighting the beneficial role of GO in enhancing electrochemical performance.

**Fig. 10:**
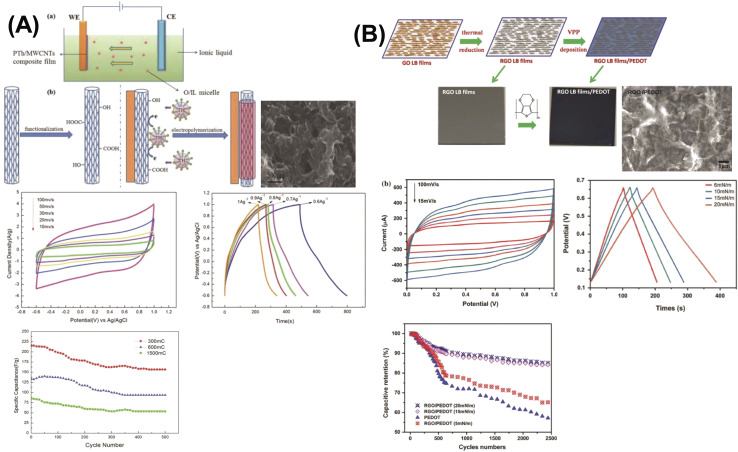
Comparison between two studies, including 1D PTh materials where one shows schematic illustrations of the formation process of PTh/MWCNT composite in an oil-in-ionic liquid micro-emulsion (O/IL) (A) and the other shows schematic illustrations of the formation process of 2D RGO LB films/PEDOT (B) and also comparing with the cross-section SEM images, CV curves at different scan rates, galvano-static charge discharge curves at different current densities and cycle stability with capacitance retention value within multidimensional devices.^90,91^ [Reproduced from ref. 90 with permission from Springer-Nature, copyright 2025 and ref. 91 with permission from The Royal Society of Chemistry, copyright 2025].

Similarly, Alvi *et al.* explored the synthesis, characterization, and electrochemical applications of a polythiophene (PTh)/graphene nanocomposite as a supercapacitor electrode.^89^ Their study indicated that the composite held strong potential in supercapacitor technology. Wen *et al.* employed the Langmuir–Blodgett technique to fabricate GO layers, followed by thermal reduction and vapor phase polymerization (VPP) of EDOT. By adjusting the deposition time, they achieved a 40 nm thick PEDOT layer atop the graphene substrate, attaining an electrical conductivity of 377.2 S cm^−1^. The cyclic voltammetry (CV) curves of PEDOT/graphene composites exhibited a rectangular shape, in contrast to the distorted CV curve of standalone PEDOT, suggesting a significant enhancement due to the presence of graphene. These nanocomposites also demonstrated a specific capacitance of 213 F g^−1^ and retained 87% of their capacity after 2000 charge–discharge cycles (Fig. 10).^90^

### 5.4 Three-dimension

Two-dimensional (2D) nanostructures are defined as sheet-like materials with a high aspect ratio. These structures typically consist of only a few atomic layers in thickness, while their other two dimensions extend beyond the nanometer scale.^76,81^ Notable examples of 2D carbon nanostructures include graphene, graphene oxide (GO), and reduced graphene oxide (rGO), which have attracted significant interest in electrochemical energy storage applications due to their exceptional surface area, conductivity, and mechanical flexibility.^76^ Du *et al.* synthesized polyaniline/graphene nanosheet (PANI/GNs) composites under various conditions and observed that the electrical conductivity of the composites increased with higher GN content.^87^ This enhancement was attributed to strong π–π interactions between GNs and PANI, where GNs acted as templates, promoting more planar and ordered PANI chain arrangements. GO, as an oxidized derivative of graphene, has also been extensively studied in combination with conducting polymers. For instance, Wang *et al.* developed a high-performance electrode material composed of fibrillar PANI doped with GO using a soft chemical approach.^98^ The resulting composite demonstrated excellent conductivity (10 S cm^−1^ at 22 °C) and a significantly enhanced specific capacitance of 531 F g^−1^ at 0.2 A g^−1^ within a potential window of 0 to 0.45 V, outperforming pure PANI (216 F g^−1^). This indicates the substantial role of GO in improving the electrochemical performance of the composite.

**Fig. 11:**
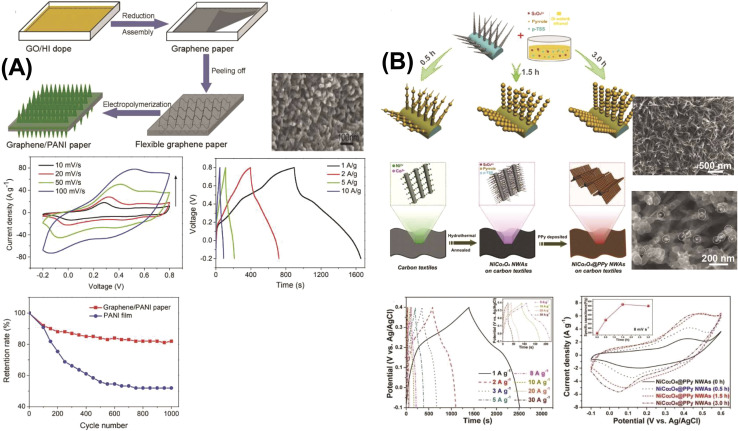
Comparison between two papers including 3D spinel structured materials where one shows schematic illustrations of the fabrication process of hierarchical mesoporous NiCo_2_O_4_@PPy hybrid NWAs on carbon textiles (B) and other shows 2D schematic illustrations of the formation process of graphene–PANI paper (A) and also comparing with the TEM and SEM images, CV curves at different compositions and times, Galvano-static charge discharge curves at different compositions and current densities, and cycle stability with capacitance retention value within multidimensional devices (Table 4).^78,92^ [Reproduced from ref. 78 with permission from Elsevier, copyright 2025 and ref. 92 with permission from The Royal Society of Chemistry, copyright 2025].

Alvi *et al.* also synthesized and characterized a polythiophene/graphene nanosheet (PTh/GN) nanocomposite for supercapacitor applications, identifying it as a promising electrode material.^89^ Similarly, Wen *et al.* fabricated PEDOT/graphene nanocomposites by depositing graphene oxide layers *via* the Langmuir–Blodgett technique, followed by thermal reduction and vapor phase polymerization (VPP) of EDOT.^90^ By adjusting the deposition time, a 40 nm PEDOT layer was formed on top of the graphene. This composite exhibited a high electrical conductivity of 377.2 S cm^−1^. The PEDOT/graphene nanocomposite showed a rectangular CV curve (indicative of ideal capacitive behavior), in contrast to the distorted shape observed with pure PEDOT, underscoring graphene's effect. The material demonstrated a specific capacitance of 213 F g^−1^ and maintained 87% capacitance retention after 2000 cycles (Fig. 11).^78^ Ternary nanocomposites are at the forefront of material innovation in hybrid capacitors, integrating the complementary features of three separate components to provide enhanced electrochemical synergy, improved ion transport, and increased charge storage efficiency.

## 6. Ranking of energy storage devices

However, a unified generic term was lacking for these devices and researchers have generally referred to them using different nomenclatures such as ‘redox capacitors’, ‘Li-ion capacitors’,^110,111^ ‘Na-ion capacitors’,^112^ ‘hybrid electrochemical capacitors’,^113^ ‘battery–supercapacitor hybrids’,^41^ or ‘pseudo-capacitors’,^114^ depending on the electrode materials and device architectures. To address this inconsistency, the generic term ‘super-capattery’ (a combination of ‘supercapacitor’ and ‘battery’) was proposed to describe these hybrid electrochemical energy storage devices that exhibit performance characteristics and operating principles distinct from both traditional supercapacitors and rechargeable batteries.

**Fig. 12:**
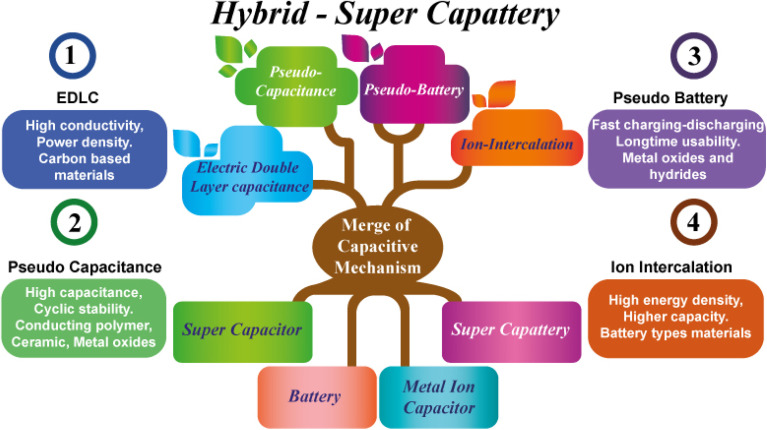
Schematic representation of different types of mechanisms that can merge owing to build up different types of energy storage devices *via* different groups of materials exhibiting different types of electrochemical energy-based properties.

The term ‘super-capattery’ was first introduced in an industrial electrochemical energy storage project initiated in 2007. Since then, its usage has gained increasing acceptance within the research community, supported by ongoing efforts to define and distinguish it based on fundamental electrochemical principles and device performance (see Fig. 12).^23,115,116^ Despite the establishment of this terminology, confusion persists in the literature.^27,117^

### 6.1 Supercapacitor

With the growing demand for clean and sustainable energy, electrochemical supercapacitors have emerged as promising energy storage and power supply devices due to their high power density, excellent efficiency, and long cycle life. Their practical feasibility has been demonstrated in various applications, including hybrid power sources, backup power systems, fuel cell starting power, and burst power generation in electronic devices.^1^ Aqib Muzaffar *et al.* highlight that the concept of double hybridization—combining nano-hybrid electrodes with activated carbon capacitive electrodes—opens new avenues for supercapacitor development and enhanced performance.^15^ However, for materials such as conductive polymers, ceramics, and certain metal oxides that exhibit high specific capacitance, a significant challenge remains: the volume changes (swelling and shrinking) during charge–discharge cycles lead to reduced supercapacitor lifespan. Electrochemical supercapacitors are a key class of hybrid storage devices, providing high power density, rapid charge–discharge capabilities, and long cycle life; yet, difficulties such as material swelling during cycling continue to limit the full potential of polymeric and ceramic-based electrodes.

### 6.2 Supercapattery

The new concept that was first proposed in 2011 is combining the best properties of super-capacitor or pseudo-capacitor with pseudo-battery types of material that is under research and development, known as super-capattery (super-capacitor + battery).^9,12,118^ This device will benefit by using two types of mechanisms that ensure high energy from battery-type material and high power from super-capacitor type material and broaden the cell voltage and extend the cell lifetime. Fundamentally, a super-capattery device integrates both capacitive and faradaic charge storage mechanisms within a single system to optimize both energy density and power density. There are four possible configurations for assembling a super-capattery:

Capacitive faradaic system + capacitive non-faradaic system (pseudo-capacitive + electric double-layer capacitor, EDLC). Capacitive faradaic system + capacitive faradaic system (pseudo-capacitive + pseudo-capacitive). Capacitive non-faradaic system + non-capacitive faradaic system (EDLC + battery). Capacitive faradaic system + non-capacitive faradaic system (pseudo-capacitive + battery). Supercapattery devices combine the principles of supercapacitors and batteries, utilizing both high power from capacitive systems and high energy from faradaic materials, resulting in variable configurations and enhanced cell voltage, energy density, and cycling life.^14,115,119^

### 6.3 Metal-ion capacitor

Pseudo-battery type electrode materials show cyclic voltammetry, charge–discharge properties close to the rechargeable battery, as discussed in the mechanism section and pseudo-capacitor type materials (conducting polymer, RuO_2_, MnO_2_*etc.*) that almost relate to super-capacitor type properties, as shown above in Fig. 12.^25,120,121^ A combination of both mechanisms can be made up metal ion capacitor. For comparison with super-capacitors, that means combined behavior is also presented for distinguishing between the devices. Some researchers classify the metal ion capacitor as a branch of super-capacitors, but it has a unique intercalation/de-intercalation mechanism that never shows super-capattery as this property is close to the battery. Super-capattery device deals only EDLC and various redox reaction (pseudo capacitor/pseudo-battery) mechanisms.^121^ By using different capacitive mechanism bearing materials, the charge–discharge profile varies significantly.^9,111,118^ Metal-ion capacitors combine the characteristics of metal-ion batteries and supercapacitors, employing asymmetric configurations that balance high energy and power densities, aided by rapid ion diffusion and reliable electrochemical interfaces.

### 6.4 Metal-ion battery

In Li-ion battery systems, lithium ions shuttle between the positive and negative electrodes during charge and discharge cycles. The primary charge storage mechanisms in Li-ion batteries are categorized into three types: intercalation, conversion, and alloying.^27,122^ Battery-type materials exhibit distinct cyclic voltammetry (CV) and galvanostatic charge–discharge (GCD) profiles characterized by pronounced current peaks near the inherent redox potentials in CV, indicating charge storage *via* reversible faradaic redox reactions. These redox processes are typically accompanied by crystal phase transitions, which manifest as voltage plateaus in the GCD curves corresponding to the coexistence of multiple phases. Unlike capacitive materials, these electrode materials predominantly store charge through bulk faradaic reactions.

**Fig. 13:**
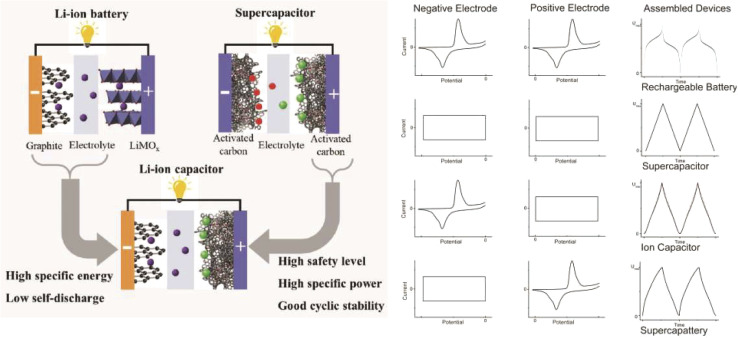
Li-ion capacitor (LIC) utilizes the best features of super capacitors with the benefits of Li-ion batteries by combining materials developed by IFE (https://www.ife.no/en/from-wood-chips-and-silicon-to-high-power/) and comparison of CV and charge–discharge profile of different types of energy storage devices.^121^ [Reproduced from ref. 121 with permission from The Royal Society of Chemistry, copyright 2025].

The energy storage mechanisms of various electrode materials can be distinctly identified through electrochemical techniques such as cyclic voltammetry (CV) and galvanostatic charge–discharge (GCD) measurements (Fig. 13). Electric double-layer capacitors (EDLC) and pseudocapacitive materials typically exhibit nearly rectangular or quasi-rectangular CV curves, respectively. Their GCD profiles show linear or slightly nonlinear voltage changes over time at a constant current. The slight nonlinearity in the GCD curve arises from the combined contributions of double-layer capacitance and pseudocapacitance. For instance, graphene, a prototypical EDLC material, displays a rectangular CV curve and linear voltage-time dependence in GCD measurements.^123^

Pseudocapacitive materials, while similar in appearance to EDLCs in CV and GCD curves, primarily store energy *via* reversible surface redox reactions involving ion insertion/de-insertion or doping/de-doping processes at the electrode–electrolyte interface, typically without inducing bulk crystal phase changes.^124^ Likewise, composites combining EDLC materials with battery-type or pseudocapacitive materials demonstrate mixed electrochemical behavior, exhibiting characteristics of both EDLC and pseudocapacitive or battery-like charge storage in their CV and GCD profiles.^14^ Finally, the hybrid super-capattery device may fulfill our required demands in a future life by inserting different types of materials as a nanocomposite, which shows a hybrid capacitive mechanism in both electrodes that may result in excellent energy density, best cyclic stability without altering power density, and voltage windows. Metal-ion batteries provide high energy density *via* reversible ion intercalation in structured electrode materials, and they are constantly changing with nanostructuring technologies to improve rate capability, capacity, and cycling durability.

Hybrid capacitive mechanisms have moved beyond laboratory studies and are now being explored in diverse real-world applications. Notable examples include their integration into flexible and wearable electronics, electric vehicles, portable power systems, and grid-level energy storage. Case studies highlight the use of graphene–polymer composites in wearable supercapacitors, metal-oxide-based pseudocapacitors in electric buses, and hybrid lithium-ion capacitors for fast-charging portable devices. These demonstrations underline the applied relevance of hybrid capacitive systems by effectively bridging laboratory-scale developments with practical device applications.

### 6.5 Theoretical perspectives and computational insights

In the past few years, significant guidance has been provided to understand and optimize the systems for hybrid supercapacitors through the utilization of computational-based modeling systems. Specifically, to investigate the electronic structure, adsorption energies, electrode material's redox activity, and to predict charge storage mechanisms at an atomic level, density functional theory (DFT) is noteworthy to use. Additionally, these aforementioned studies can also be complemented by using molecular dynamics (MD)-based modeling to investigate the behavior of ion transportation, effects of solvation, and interfacial dynamics of electrode–electrolyte under different operating conditions.^125^ Furthermore, the combination of continuum and kinetic Monte Carlo models has also offered insights into the kinetics of diffusion, distribution of charge, and long-term stability. Mutually, all of these theoretical approaches reduce the gap between structure and performance, and simultaneously correlate the properties of different materials along with electrochemical experimental-based outcomes. These outcomes are also vital to guide numerous factors, which include the flexibility of a rational design, boosting of hybrid electrodes; performance, and the gradual elimination of the trial-and-error approach during synthesis.^126,127^ Thus, utilizing an integrated computational modeling system can augment the rapid development of next-generation technologies for energy storage.

### 6.6 *In situ* and *operando* spectroscopic studies

To understand electrochemical mechanisms, characterizations of *in situ* and *operando* spectroscopy are the critical factors that involve examining structural and electronic changes during actual device operation. However, to track the variations of the crystal lattice as well as the transition of phases during the process of ion intercalation, *in situ* X-ray diffraction (XRD) is noteworthy to use. Likewise, worthy information in terms of redox states and the environments of local bonding in metal oxides and pseudocapacitive polymers can also be obtained from *operando* Raman and infrared (IR)-based spectroscopic analysis.^32^

In addition to XRD and IR spectroscopy, X-ray photoelectron spectroscopy (XPS) analysis is also an effective method to identify transition metals' valence state variations, which are directly related to both redox chemistry and capacitance behavior [Ref]. Moreover, the visualization of morphological evolution is also very much possible using a combination of transmission electron microscopy (TEM) and electrochemical cells. This advancement is simultaneously shedding light on different associated mechanisms of degradation alongside the expansion of volume in electrode materials. The application of electrochemical impedance spectroscopy (EIS) further facilitates the study of resistance correlation, diffusion, and dynamics of charge-transfer with applied potentials. Together, a groundbreaking advancement of characterization methods provides crucial mechanistic insights, which cannot be retrieved by *ex situ* studies only. They are also playing a pivotal role in minimizing the gap between materials discovery and practical optimization of the devices.^30^

## 7. Future perspective and challenges

Electrochemical energy storage devices are the critical technologies that have made themselves remarkable in terms of high-power density, efficiency, along with long cycle life. In addition, they are also useful to find efficient hybrid power systems, backup supplies, fuel cell startups, and portable electronics. It is also vital to optimize electrode materials in conjunction with a high specific surface area, compatible pore architectures for the rapid transportation of ions, low internal resistance, and strong electrochemical/mechanical stability for the assurance of cyclic durability. In this case, nanostructured electrodes, which include a range of materials such as aerogels, nanotubes, and nanosheets, accompanied by composite architectures, can offer synergistic benefits. Particularly, enhanced conductivity, expanded active sites, and improved mechanical integrity can be ensured using these aforementioned composite nanostructured electrodes. Computational-based modeling is also very helpful for integrating a rational design of such materials with the combination of the electrode's structure, performance, and guiding optimization strategies.^128^

Numerous challenges still exist, which hinder the deployment of hybrid energy storage systems on a larger scale. Precisely, the rapid degradation of pseudocapacitive-based electrode materials is still posing a challenge because of their volumetric changes at the time of cycling. The stability of the electrolyte needs to be improved for wide potential windows, while ensuring safety and environmental compatibility are also vital to consider. However, in terms of existing challenges, the scalability and cost-effective approach to fabricating nanostructured composites also remain pressing concerns. Because, in real-life applications, it's not that easy to replicate laboratory successes industrially. Likewise, a successful integration into flexible and multifunctional devices also demands such materials, which can balance electrochemical performance along with mechanical strength.^126^

Overall, future research regarding electrochemical energy storage should focus on several points, such as (i) hierarchical nanostructures that synergize EDLC and pseudocapacitive behaviors; (ii) multifunctional composites with the combination of carbons, polymers, and ceramics; (iii) synthesis routes, which can confirm both eco-friendliness, and scalability; and (iv) advanced *in situ*/*operando* characterization methods as well as computational modeling for the acceleration of materials discovery. Therefore, if it's likely to address all of these aspects, it would be possible to translate hybrid capacitive mechanisms from laboratory innovation into sustainable and commercially viable energy storage technologies.^95^

## 8. Conclusion

Energy storage devices are recognized as pivotal solutions for sustainable and renewable energy applications ranging from electronics to large industrial machinery. Nanostructured electrode materials, particularly nanoporous architectures and composites, enhance ion and electron transport, specific capacitance, and device durability. This study summarizes key materials and mechanisms for hybrid energy storage, emphasizing the impact of conducting polymers, carbon composites, complex ceramics, and metal oxides/hydrides, supported by morphological and structural features. The insights this review provided offer future directions on developing high-performance energy storage devices and nanocomposite materials.

## Author contributions

Salman Farsi: Investigation; writing – original draft. Mushfiqur Rahman: Investigation; writing – original draft. Thuhin K. Dey: Investigation; writing – original draft. A. J. Saleh Ahammad: Writing – review & editing. Mamun Jamal: Conceptualization (lead); supervision; writing – original draft (lead); writing – review & editing (lead).

## Conflicts of interest

There are no conflicts to declare.

## Data Availability

No data were generated in this study.
